# Peptide immunization against the C-terminal of alpha-synuclein reduces locomotor activity in mice overexpressing alpha-synuclein

**DOI:** 10.1371/journal.pone.0291927

**Published:** 2023-09-21

**Authors:** Yu-Sung Chiu, Kuo-Jen Wu, Seong-Jin Yu, Kun-Lieh Wu, Yu-Syuan Wang, Jing Lin, Chia-Ying Chu, Shuchun Chen, Hsi Chen, Shu-Ching Hsu, Yun Wang, Yun-Hsiang Chen

**Affiliations:** 1 Department of Life Science, Fu-Jen Catholic University, New Taipei City, Taiwan; 2 National Health Research Institutes, Center for Neuropsychiatric Research, Zhunan, Taiwan; 3 School of Pharmacy, College of Pharmacy, China Medical University, Taichung, Taiwan; 4 Department of Electrical Engineering of I-Shou University, Kaohsiung, Taiwan; 5 Institute of Infectious Diseases and Vaccinology, National Health Research Institutes, Zhunan, Taiwan; 6 Graduate Institute of Medicine, College of Medicine, Kaohsiung Medical University, Kaohsiung City, Taiwan; 7 PhD Program in Tissue Engineering and Regenerative Medicine, National Chung Hsing University, Taichung City, Taiwan; 8 Graduate Institute of Biomedical Science, China Medical University, Taichung City, Taiwan; 9 Immunology Research and Development Center, China Medical University, Taichung City, Taiwan; 10 Department of Life Sciences, Tzu Chi University, Hualien, Taiwan; Louisiana State University Health Sciences Center, UNITED STATES

## Abstract

Abnormal accumulation of alpha-synuclein (αSyn) in the remaining nigra dopaminergic neurons is a common neuropathological feature found in patients with Parkinson’s disease (PD). Antibody-based immunotherapy has been considered a potential approach for PD treatment. This study aims to investigate the effectiveness of active immunization against αSyn in a mouse model of PD. Adult mice were immunized with or without a synthetic peptide containing the C-terminal residues of human αSyn and activation epitopes, followed by an intranigral injection of adeno-associated virus vectors for overexpressing human αSyn. Upon the peptide injection, αSyn-specific antibodies were raised, accompanied by degeneration of dopaminergic neurons and motor deficits. Furthermore, the induction of neuroinflammation was postulated by the elevation of astroglial and microglial markers in the immunized mice. Instead of lessening αSyn toxicity, this peptide vaccine caused an increase in the pathogenic species of αSyn. Our data demonstrated the potential adverse effects of active immunization to raise antibodies against the C-terminal fragment of αSyn. This drawback highlights the need for further investigation to weigh the pros and cons of immunotherapy in PD. Applying the αSyn C-terminal peptide vaccine for PD treatment should be cautiously exercised. This study provides valuable insights into the intricate interplay among immune intervention, αSyn accumulation, and neurodegeneration.

## Introduction

Parkinson’s disease (PD) is the second most common neurodegenerative disorder, affecting 1–2% of the population over 65 years old [1]. The typical pathological features of PD are the progressive loss of dopamine-producing neurons in the substantia nigra pars compacta and intracellular alpha-synuclein (αSyn) aggregation in the remaining neurons [2]. As current treatments for PD mainly provide symptomatic relief but do not ameliorate the degeneration of dopaminergic neurons [3], alternative therapeutics to alter the progression of neurodegeneration are in demand.

αSyn is an intracellular protein of 140 amino acid residues predominantly expressed in presynaptic terminals throughout the central nervous system. Although the physiological role of αSyn remains unclear, it may regulate synaptic activity such as neurotransmitter release and synaptic plasticity [4–6]. Abnormal accumulation of αSyn, especially the oligomeric αSyn, in axons and presynaptic terminals promotes the degeneration of dopaminergic neurons [7–9]. The formation of αSyn oligomers is affected by modulating the synthesis, aggregation, or degradation of αSyn [10,11]. Oligomeric αSyn is predominantly secreted from the neuronal cytoplasm through nonclassical exocytosis, independent of the ER-Golgi pathway [12]. The extracellular oligomers are internalized by neighboring neurons and glial cells, leading to aggregate propagation in a prion-like fashion [12–16]. Extracellular and intracellular αSyn have become promising targets for antibody-based immunotherapy, accelerating the clearance of oligomeric αSyn and preventing dopaminergic degeneration in PD animals [17–20].

αSyn immunotherapy can be achieved through passive or active immunization. In passive immunization, the administration of a monoclonal antibody against the C-terminal residues (118–126) of human αSyn reduced αSyn accumulation and ameliorated behavioral and neuropathological deficits in the αSyn-transgenic mice [21]. Given that the C-terminal cleavage of αSyn and the interaction of C-terminal fragments with full-length αSyn are involved in the formation of αSyn aggregates [22,23], the C-terminal antibody was suggested to block the cleavage of αSyn at amino acid residues 121–123 by calpain protease [24] and therefore reduce αSyn aggregations [21]. However, passive immunization did not provide a steady state antibody level. Repetitive infusion is required to maintain effective antibody titers. Active immunization was achieved by using a non-pathogenic portion of the antigen, such as the C-terminal peptide of αSyn. Synthetic linear peptides are often used as immunogens to elicit antibody responses against the selected epitope and are easy for large-scale manufacturing. Peptide-based vaccines require the conjugation with carrier proteins to provide MHC-II epitopes and induce a crosslink of surface immunoglobulin receptors to activate helper T lymphocytes and peptide-specific B cells [25,26]. One major limitation of this approach is that carrier proteins may induce carrier-specific antibodies or regulatory T cells, and the rejection of the vaccines [27–30].

**Fig 1:**
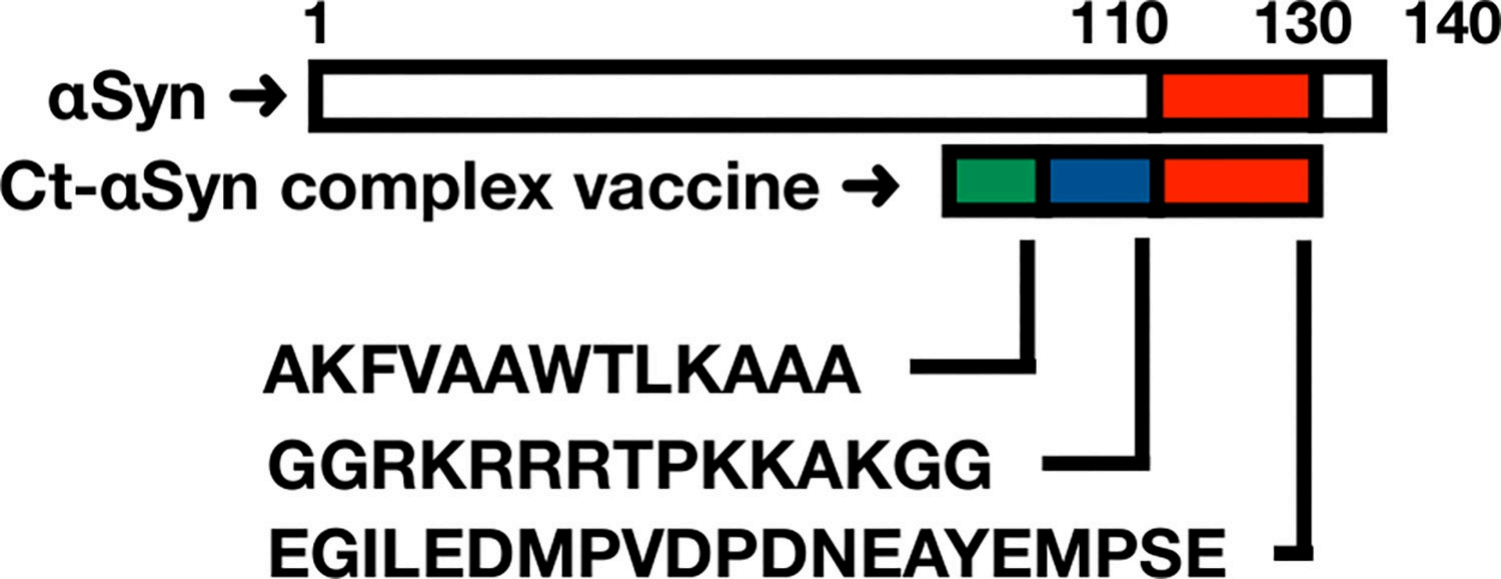
Schematic illustration of the Ct-αSyn complex vaccine. A linear peptide consists of a pan DR T helper epitope (AKFVAAWTLKAAA; green), an arginine-rich motif (GGRKRRRTPKKAKGG; blue) derived from the Tat protein of simian immunodeficiency virus, and a peptide corresponding to amino acid residues 110–130 of human αSyn (EGILEDMPVDPDNEAYEMPSE; red). This combination peptide was adjuvanted with alum salts and used as a Ct-αSyn complex vaccine to immunize mice in this study.

Therefore, we designed a novel peptide vaccine formulated in adjuvant alum without carrier conjugation to avoid high immune rejection from the carrier. The synthetic C-terminal peptide complex (Ct-αSyn complex) is composed of a short peptide (corresponding to amino acid residues 110–130 of human αSyn) covalently linked to a pan DR T helper epitope (PADRE) and an arginine-rich motif (Fig 1). PADRE is a non-natural peptide binding with high or intermediate affinity to several common HLA-DR and mouse MHC class II alleles, capable of stimulating helper T cells to induce antibody responses to the linked peptide [31,32]. In addition, the arginine-rich motif, derived from the simian immunodeficiency virus (SIV) Tat protein, is able to amplify antibody responses to its fusion protein, although the mechanism of action is unknown [33].

This study investigated the outcome of active immunization with Ct-αSyn complex in a mouse PD model overexpressing human αSyn in the substantia nigra [34,35]. We found that the Ct-αSyn complex promoted the production of antibodies targeting the C-terminal region of human αSyn, but exacerbated αSyn-induced motor dysfunction accompanied by increased αSyn accumulation and neurotoxicity. Our data raise concerns about potential neuronal damage attributed to immunotherapy based on active immunization against αSyn.

## Materials and methods

### Cell cultures

The human embryonic kidney cell line (HEK293; #240073, Agilent Technologies) was grown as monolayers in Dulbecco’s modified Eagle’s medium (DMEM) supplemented with 10% heat-inactive fetal calf bovine serum (FBS), and antibiotics [streptomycin (100 μg/ml) and penicillin (100 IU/ml)] at 37°C in a humidified incubator with 5% CO_2_.

### Synthesis of Ct-αSyn complex

A linear peptide (Ct-αSyn complex; Fig 1) composed of a pan-DR T helper epitope (PADRE: AKFVAAWTLKAAA), an arginine-rich motif (GGRKRRRTPKKAKGG) derived from the simian immunodeficiency virus (SIV) Tat protein, and an αSyn C-terminal region (αSyn_110–130_: EGILEDMPVDPDNEAYEMPSE) was synthesized with purity greater than 80% by Genomics Ltd. This synthetic peptide was adsorbed onto alum salts to immunize mice, as described in our previous work [33]. Briefly, 5 ml of PBS, 500 μl of 1M NaHCO_3_, and 1 ml of 10% alum [AlK(SO_4_)_2_] were thoroughly mixed to form a milky solution. After centrifugation at 10,000 ×g at room temperature for 5 min, the precipitated pellet was resuspended in 5 ml of PBS (containing 1,000 μg Ct-αSyn complex), followed by incubation on ice for 1 hour.

### Plasmid construction

The plasmid pAAV-αSyn (kindly provided by Dr. Brandon K. Harvey, OTTC, NIDA, USA) used for the production of adeno-associated virus (AAV) particles was constructed as previously described [19]. Briefly, the cDNA of human αSyn, fused to a V5-tag coding sequence at the 5’-end, was cloned into an AAV shuttle plasmid (pAAV) at KpnI and EcoRV restriction sites. This shuttle plasmid contains the following genetic fragments aligned in order: the left inverted terminal repeat (L-ITR) of AAV2, EF1-α promoter, multiple cloning sites, post-transcriptional regulatory element of the woodchuck hepatitis B virus (WPRE), polyadenylation signal of human growth hormone (hGH polyA), and right inverted terminal repeat (R-ITR) of AAV2.

### Virus production and purification

As previously described, the AAV particles (AAV1-αSyn) were generated by triple plasmid transfection [20,36]. In each culture dish (15-cm diameter), HEK293 cells (70–80% confluent) were cotransfected with a vector plasmid (pAAV-αSyn; 9 μg), a capsid plasmid (pRC1; 3.5 μg), and a helper-adenovirus plasmid (pHelper; 12.5 μg; Agilent Technologies) by the TransIT-X2 reagent (75 μl; #MIR6003, Mirus) as per manufacturer’s recommended protocol. At 48–58 hours after transfection, cell pellets were harvested by centrifugation (2,500 ×g at 4°C; the same setting used in the following centrifugation procedures) for 10 min and resuspended in the suspension buffer (50 mM Tris-HCl pH8.0, 150 mM NaCl, 2 mM MgCl_2_; 1 ml buffer for a cell pellet in a culture dish). The resuspended cells were burst by freezing (-80°C for 30 min) and thawing (37°C for 15 min) for three rounds. After centrifugation for 20 min, the supernatants of cell lysates were collected. On the other hand, the culture media of transfected cells were mixed thoroughly with 40% PEG8000 (in 150 mM NaCl; #1546605, Merck) to a final concentration of 8% and incubated at 4°C for two hours. The mixtures were centrifuged for 20 min, and the precipitated pellets were dissolved in the suspension buffer at 1/80 of the starting volume. Finally, this resuspension and the supernatant of cell lysates were combined and supplemented with Triton-X-100 (1%) and Benzonase (100 U/ml; #E1014, Sigma). After incubation at 37°C in a water bath for one hour, the mixture was centrifuged for 20 min, and the supernatant was filtered through a 0.2 μm filter cup. AAV particles were purified using the HiTrap AVB affinity column (#GE28-4112-11, Cytiva) equipped with a P1 peristaltic pump (#18111091, Cytiva) set at a flow rate of 2 ml/min. After equilibrating with 20 ml of PBS, the column was loaded with the filtered supernatant (containing AAV particles) and washed with 20 ml of PBS. AAV particles were eluted from the column with 8 ml of glycine buffer (50 mM, pH2.7), and every elution fraction (1 ml) was collected in a microtube (containing 100 μl of 1M Tris-HCl, pH8.0). The collected fractions with an O.D.280 value over 0.2 were pooled together and concentrated in Spin-X UF500 filter tubes (100,000 MWCO; #431481, Corning) by centrifugation until the volumes were reduced by 90%. After that, the filter tube was filled with PBS and centrifuged again, as described above. The concentrated viral suspension was filtered through a 0.2 μm filter disk, aliquoted (15 μl/vial), and stored at -80°C until use.

### Virus titration

The titer of purified AAV particles was determined by the quantitative real-time polymerase chain reaction (qPCR) performed on an ABI StepOnePlus system as previously described [20,36,37]. The purified virus sample (5 μl) was pretreated with two units of DNase I (#M0303, New England BioLabs) in a reaction volume of 50 μl at 37°C for 1 hour, followed by incubation at 75°C for 10 min to inactivate DNase I. A pair of primers (forward: 5’- TCATGCTATTGCTTCCCGTATGG -3’; backward: 5’- GGATTGAGGGCCGAAGGGA -3’), targeting the WPRE region on the vector plasmid (pAAV-αSyn), were designed by the Primer-3 program to amplify a 384-bp fragment in qPCR reactions. Each qPCR reaction mixture (20 μl) contained 2 μl of DNase-pretreated virus sample, 10 μl of 2× SYBR green PCR master mix (#4367659, ABI), and 0.5 μM of each primer. Each reaction was triplicated in every qPCR assay. The PCR cycling program was set as the following: 95°C for 10 min, followed by 40 cycles for amplification (95°C for 15 sec, and 60°C for 30 sec), and a cycle for generating a melting curve (95°C for 15 sec, 60°C for 1 min, and 95°C for 15 sec). Along with each virus titration, a standard curve (plasmid copy number versus cycle threshold value) was generated using ten-fold serially diluted (0.01–100 pg) pAAV-αSyn plasmids as templates in the qPCR assay. Based on this standard curve, virus titers were calculated and expressed as viral genome copies per milliliter of the virus sample (VGC/ml).

### Animals

Adult male C57BL/6 mice (3-month-old) were purchased from BioLASCO Co., Ltd., and group housed under environmentally controlled conditions (lights on at 7:00 a.m. and lights off at 7:00 p.m.; 24 ± 2°C constant room temperature; 50 ± 10% relative humidity). Animals were allowed to habituate for two weeks after their arrival and had free access to autoclaved food and drinking water. Animal experiments were conducted under the animal protocol reviewed and approved by the Animal Research Ethics Board at National Health Research Institutes in Taiwan (Permit Number: NHRI-IACUC-111054-A). The ARRIVE guidelines were used for the animal experiments. Animals were numbered randomly using the RAND function in Mac*’s Numbers* application for allocation to control and treatment groups. The researchers who conducted the treatment were aware of the group allocation. The animal care staffs and the researchers analyzing the data were unaware of the group allocation.

### Immunization with Ct-αSyn complex

Mice were immunized with alum-adjuvanted Ct-αSyn complex (100 μg/500 μl/mouse; n = 7) or PBS (n = 14) via intraperitoneal injection on weeks 0, 2, 4, 6, and 17. In addition, blood samples were collected from tail veins on days -2 and 60, and sera were subjected to immunofluorescence staining and ELISA tests to determine the specificity and titers of antibodies against αSyn.

### Intracranial injection of AAV1-αSyn

On week 10, after receiving the first intraperitoneal injection of peptide vaccine or PBS, mice were intracranially injected with AAV1-αSyn to overexpress human αSyn in the brain. Briefly, mice were anesthetized by intraperitoneal injection of pentobarbital (35 mg/kg) and then placed on a stereotaxic frame. After a burr hole was drilled in the skull, mice were injected with AAV1-αSyn (1 μl, 1.0 × 10^10^ VGC/μl) or vehicle (PBS, 1 μl) into the right substantia nigra (AP: -2.9 mm from the bregma; ML: -1.3 mm from the midline; DV: -4.2 mm from the dura). The virus was delivered by a Hamilton Microliter syringe (10 μl volume, cemented needle, 22s gauge, blunt tip) controlled by a microprocessor at an infusion rate of 0.2 μl/min. After completing the infusion step, the needle was kept in the brain for 2 min and then slowly removed. A piece of bone wax was placed on the burr hole to prevent fluid leakage, and the wound was sutured. The anesthetized state is monitored by observing the respiration rate and reflexes of the tail pinch, toe pinch, and eye blink. During recovery from anesthesia, animals were placed on a heating pad in an environmental chamber with an airflow set to maintain the animal’s body temperature at 37°C. After recovery from the anesthesia, animals were sent to their home cages and monitored daily for wound healing and food/water intake. To relieve the pain, we injected (SC) the animals with buprenorphine (0.03 mg/kg BW) for three consecutive days after the surgery.

### Measurement of locomotor activity

Mice were individually placed in the activity chambers (42 × 42 × 30 cm; length × width × height), equipped with infrared sensors aligned in the horizontal and vertical directions (AccuScan Instruments, Inc.). After the animals were habituated for 30 min, locomotor activities were recorded for 2 h during the light cycle (1 p.m. to 3 p.m.). Six parameters were measured, including total distance traveled (TOTDIST; distance, measured in centimeters, traveled by the animals), horizontal activity (HACTV; the number of bean interruptions detected by the horizontal sensors), vertical activity (VACTV; the number of bean interruptions detected by the vertical sensors), movement numbers (MOVNO; the counts of horizontal movements followed by a break lasting over one second), movement time (MOVTIME; the total time, counted in seconds, when the animal was detected moving), and rest time (RESTIME; the total time, counted in seconds, when the animal was detected resting).

### Immunofluorescence staining analysis

HEK293 cells were grown on 12-mm glass coverless in 24-well plates (2×10^5^ cells/well) overnight and then transfected with or without pAAV-αSyn (0.5 μg/well) using the TransIT-X2 reagent (#MIR6003, Mirus). The following procedures were conducted at room temperate, and reagents were prepared in 1× PBS. At 24 h post-transfection, cells were washed once with PBS, fixed with 4% formaldehyde for 10 min, and then permeabilized with 0.3% Triton X-100 for 10 min. After blocking with 4% FBS for 30 min, cells were incubated with the mouse monoclonal antibody Syn211 (1:200; #ab80627, Abcam) or mouse serum (1:200; collected before or after vaccination) for 1 h. Of note, when the binding competition was performed to determine whether vaccine-induced antibodies selectively recognized the C-terminal of human αSyn, mouse sera collected after vaccination were supplemented with αSyn_110–130_ peptide or Spike-binder (SALEEQYKTFLDKFMHELEDLLYQLAL) [38] at concentrations of 0.7 and 0.07 μg/μl. Cells were then washed with PBS three times for 5 min each and incubated with Alexa-Fluor-488-conjugated goat anti-mouse IgG polyclonal antibody (1:200; #GTX213111-04, GeneTex) for 1 h. After washing with PBS three times for 5 min each, cells were fixed again with 4% formaldehyde for 10 min, followed by washing with distilled water. Finally, each coverslip was lowered onto a drop of mounting medium (containing DAPI, 1.5 μg/ml; #H-1200; Vector Laboratories) on glass slides and examined by a fluorescence microscope equipped with an LED-light source module (#SLF140-390/440/470/525, SCOPELED) and an image-capturing system SGcapture 5.1.1 (#SGHD-3600, SAGE Vision).

### Measurements of peripheral αSyn and biomarkers

Peripheral blood samples were collected on days -2 (pre-Vac; 2 days before the first vaccination) and 60 (post-Vac: 18 days after the fourth vaccination). Approximately 100 μl of whole blood was collected from the tail vein of vaccinated mice and kept at room temperature for 30 min to enable clotting. After centrifugation at 10,000 ×g at room temperature for 10 min, the serum in the upper layer was collected and kept at -20°C until use. The collected sera were diluted and subjected to the measurement of peripheral αSyn by using the Alpha-synuclein SimpleStep ELISA kit (#ab282865, Abcam), 20 inflammation-related cytokines by using the Quantibody® Mouse Cytokine Array 1 (#QAM-CYT-1, RayBiotech), and lactate dehydrogenase (LDH) by using the CytoTox 96® Non-radioactivity Cytotoxicity Assay (#G1780, Promega), according to manufacturer’s instructions.

### Antibody titration

Unless otherwise stated, the following procedures were performed at room temperature, the reagent volumes for incubation were 50 μl per well, and the buffer volumes for washing steps were 200 μl per well. ***Coating plates with synthetic peptides*:** Poly-l-lysine (PLL, 30–70 kDa; #P2636, Sigma) was dissolved in 50 mM sodium bicarbonate at 40 μg/ml and added to 96-well plates. After incubation for 1 h, the plates were washed with PBS, followed by incubation with 1% glutaraldehyde (in PBS; #49629, Sigma) for 15 min. After washing with PBS once, the plates were incubated with αSyn_110–130_ peptide (10 μg/ml) at 4 ˚C overnight and then washed with PBS twice. After that, the plates were incubated with 1 M glycine (200 μl/well) for 1 h, followed by washing with PBS twice. ***Enzyme-linked immunosorbent assay*:** The peptide-coated plates were blocked with 3% skim milk (in PBS) for 1 h. After removing the milk, the plates were incubated with 2-fold serially diluted serum or antibodies (in 3% skim milk) for 1 h, followed by washing with 0.1% Tween-20 (in PBS) three times. After that, the plates were incubated with HRP-conjugated anti-mouse IgG antibodies (1:2000 in 3% skim milk; #GTX213111-01, GeneTex) for 1 h and then washed three times as described above. Finally, the plates were incubated with TMB substrate (#TM4500, ScyTek) for 10 min, the reaction was arrested by adding 1 M HCl, and the optical density (OD) was measured at a wavelength of 450 mm. The antibody endpoint titer was defined as the reciprocal of the highest serum dilution that gives an OD_450_ value 3-fold above the background value. For the undetectable sample, the antibody endpoint titer was arbitrarily defined as 10°.

### Western blotting analysis

Lysates of cultured cells and mouse brain tissues were prepared for Western blotting analysis as previously described [19,39]. For examining cell lysates, HEK293 cells were grown in 24-well plates (2×10^5^ cells/well) overnight and then transfected with or without the plasmid (pAAV-αSyn; 0.5 μg/well) encoding human αSyn using the TransIT-X2 reagent (#MIR6003, Mirus). At 24 h post-transfection, cells in each well were washed once with PBS and lysed in 100 μl of 2× Laemmli buffer (4% SDS, 125 mM Tris-HCl pH6.8, 10% b-mercaptoethanol, 20% glycerol, and 0.004% bromophenol blue in distilled water). Cell lysates were passed through a 25-gauge needle 20 times to reduce viscosity. For examining mouse brain samples, each collected brain tissue (substantia nigra or striatum) was homogenized in 100 μl of RIPA lysis buffer by vibrating for 30 sec twice using a bead mill homogenizer at 4˚C. After that, samples were centrifuged at 12,000 ×g at 4˚C for 15 min, and the supernatant was mixed with 100 μl of 2× Laemmli buffer. The Laemmli buffer-treated samples were heated at 90˚C for 10 min and subjected to electrophoresis separation on 12% SDS-polyacrylamide gels. The separated proteins were electrophoretically transferred from the gel to a PVDF membrane (#RPN303F, GE Healthcare). The membrane was then incubated with 10 ml blocking buffer (5% skim milk and 0.1% Tween-20 in PBS) for 1 h and then incubated with appropriate primary antibodies in 3 ml blocking buffer (in a 50 ml centrifuge tube) at 4˚C overnight with gentle rotation. After washing with 0.1% Tween-20 (in PBS) three times for 10 min each, the membrane was incubated with horseradish peroxidase (HRP)-conjugated goat polyclonal antibodies against mouse IgG (1:2,500; #GTX213111-01, GeneTex) or rabbit IgG (1:5,000; #GTX213110-01, GeneTex) in 3 ml blocking buffer for 1 h, followed by the washing procedure described above. The light emission signals of target proteins on the membrane were generated using an enhanced chemiluminescence reagent (##GTX14698, GeneTex) and captured by the CCD camera-based image system MultiGel-21 (TOPBIO, Taiwan). The intensity of the detected images was quantified by ImageJ software (http://imagej.gov/ij).

Five mouse monoclonal antibodies against the C-terminal of human αSyn (Syn211; 1:2,000; #ab80627, Abcam), αSyn (Clone-42; 1:1,000; #610787, BD; used to probe total αSyn), tyrosine hydroxylase (1:2,500; #MAB318, Millipore), V5 tag (1:2,000; #GTX42525, GeneTex; used to probe V5-tagged human αSyn), and β-actin (1:2,500; #GTX629630, GeneTex), four rabbit polyclonal antibodies against GFAP (1:2,500; #GTX108711, GeneTex), PSD95 (1:2,500; #GTX133091, GeneTex), Synaptophysin (1:2,500; #GTX100865, GeneTex), and Ser-129 phospho-αSyn (1:1,000; #GTX50222, GeneTex), and one rabbit monoclonal antibody against Iba1 (1:500; #01919741, Fujifilm Wako) were used as primary antibodies. Mouse sera collected before and after vaccination were diluted (1:2,000) in the blocking buffer and used as primary antibodies to probe human αSyn. Of note, the skim milk content in the blocking buffer was replaced with BSA to reduce nonspecific binding to the antibody against Ser-129 phospho-αSyn.

### Statistical analysis

All data were expressed as means ± standard error (SEM) and analyzed by GraphPad Prism 9.2.0 statistical software (GraphPad Software, Inc.). Comparisons of the mean values of proteins detected by Western blots between the two groups were performed by the unpaired, two-tailed Student’s t-test. The differences in the mean values of the antibody, αSyn, lactate dehydrogenase (LDH), and cytokine levels in the sera collected before and after vaccination were determined by the paired, two-tailed Student’s t-test. In addition, comparisons for the effect of treatment on locomotor activity were assessed by ordinary one-way analysis of variance (ANOVA) and Tukey’s post hoc test. Differences between groups are considered statistically significant when the p-value is <0.05.

## Results

### Ct-αSyn complex vaccine promoted the production of anti-αSyn antibodies

**Fig 2:**
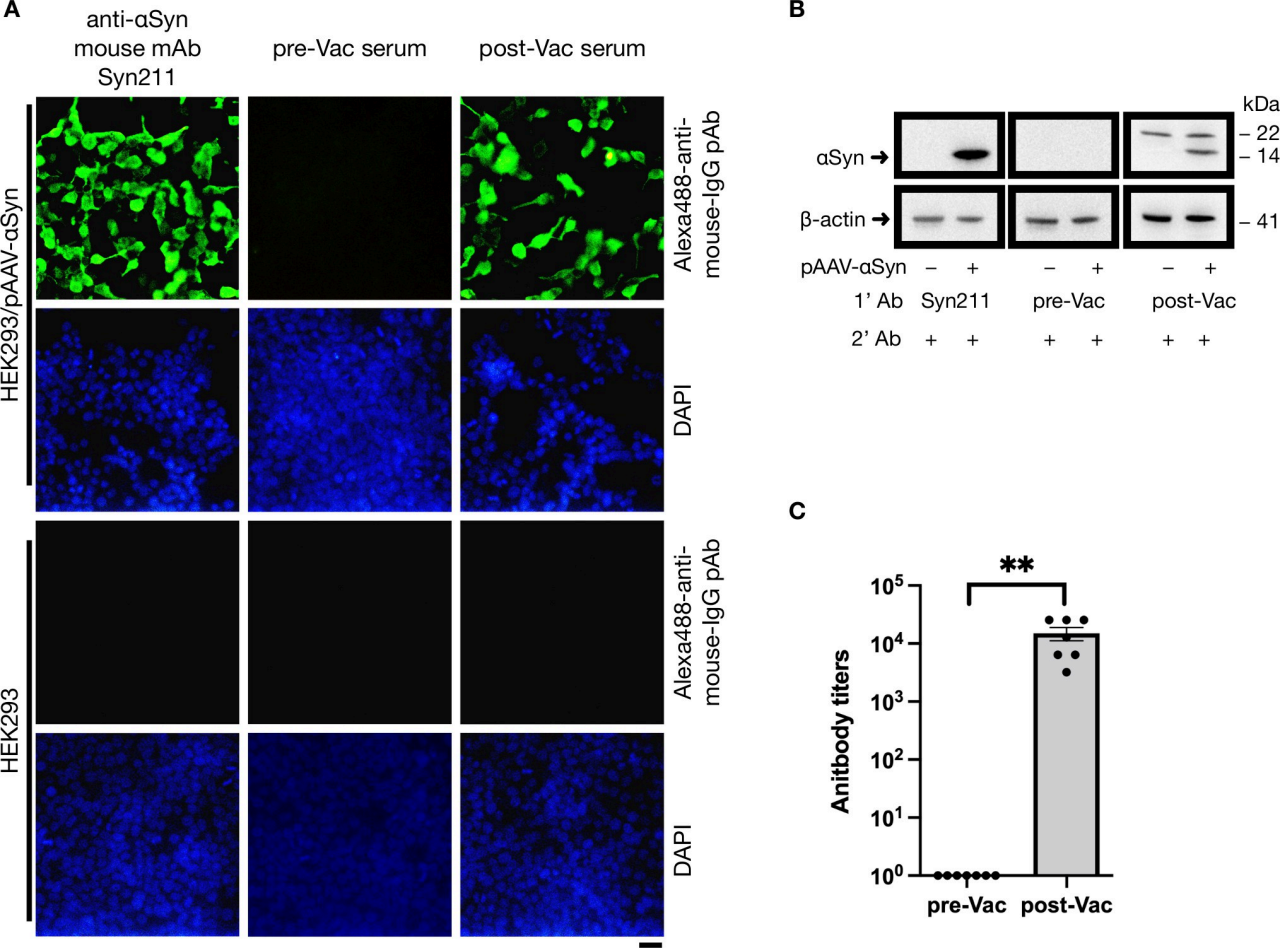
Ct-αSyn complex vaccine induces antibodies to recognize αSyn. Adult mice (n = 7) were intraperitoneally injected with Ct-αSyn complex vaccine four times at 2-week intervals. Sera were collected from tail veins before vaccination (pre-Vac) and after the fourth vaccination (post-Vac). Pooled sera and an αSyn-specific mouse monoclonal antibody (Syn211) were used as primary antibodies (1’ Ab) to probe αSyn in HEK293 cells transfected with or without pAAV-αSyn plasmids (encoding human αSyn) by (A) immunofluorescence staining and (B) Western blot analyses. In the immunofluorescence staining, Alexa488-conjugated polyclonal antibodies were used as secondary antibodies (green); cellular nuclei were labeled with DAPI (blue); scale bar = 20 μm. In the Western blot analysis, HRP-conjugated anti-mouse IgG antibodies were used as secondary antibodies (2’ Ab); the migration molecular weights of αSyn (14 kDa) and β-actin (41 kDa) were indicated. (C) All serum samples were 2-fold serially diluted for antibody titration by enzyme-linked immunosorbent assay conducted in microplates coated with αSyn_110-130_ peptide. The antibody titer was defined as the reciprocal of the highest serum dilution that gives an OD_450_ value 3-fold higher than the background value. Data are expressed as mean values ± SEM. Significant differences between groups are indicated (**p < 0.01; Student’s *t*-test).

**Fig 3:**
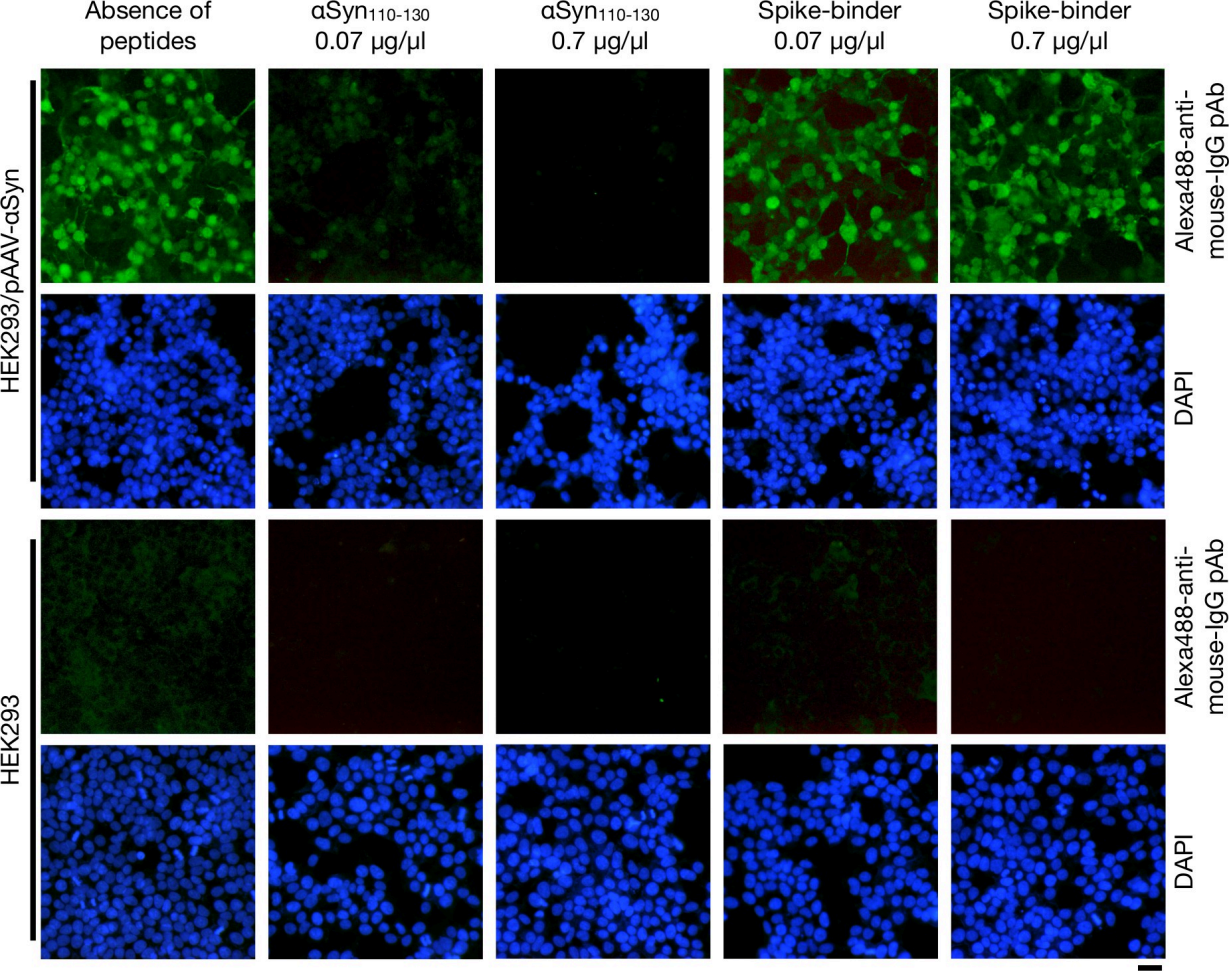
αSyn C-terminal peptides disrupt the interaction between vaccine-induced antibodies and αSyn. Adult mice (n = 7) were administered with Ct-αSyn complex vaccine via intraperitoneal injection four times at 2-week intervals. Sera were collected from tail veins after the fourth vaccination. By immunofluorescence staining analysis, pooled sera were examined for their specificity to αSyn in cells (HEK293/pAAV-αSyn) transfected with pAAV-αSyn plasmids (encoding human αSyn) and naive cells (HEK293) in the presence of αSyn_110–130_ or Spike-binder peptides at different concentrations (0, 0.7, 0.07 μg/μl). Cellular nuclei were labeled with DAPI (blue); Alexa488-conjugated polyclonal antibodies were applied to detect mouse IgG (green); scale bar = 20 μm.

Adult mice (n = 7) were intraperitoneally injected with the Ct-αSyn complex vaccine (Fig 1). Sera were collected from the tail vein before the first vaccination (pre-Vac) and after the fourth vaccination (post-Vac). The production of antibodies against αSyn was indirectly measured by immunofluorescence staining on human HEK293 cells transfected with pAAV-αSyn (encoding human αSyn) (Fig 2A). As seen in the positive control group, αSyn was detected as green fluorescence signals in the transfected cells (HEK293/pAAV-αSyn) stained with an anti-αSyn mouse monoclonal antibody (Syn211; primary antibody) and an Alexa488-conjugated anti-mouse IgG polyclonal antibody (secondary antibody). In the experimental groups, the collected sera were used as primary antibodies to probe αSyn in HEK293/pAAV-αSyn cells, where green fluorescence signals were detected by post-Vac sera but not pre-Vac sera. For all tested primary antibodies, the green fluorescence signal was not found in the naive cells (HEK293). In the Western blotting analysis (Fig 2B), αSyn (14 kDa) was detected by Syn211 and post-Vac sera but not pre-Vac sera in the cell lysate of HEK293/pAAV-αSyn. In contrast, the 14-kDa band was not detected by all three primary antibodies in naive cell lysates. These data indicate that intraperitoneal injection of Ct-αSyn complex vaccine induced antibodies against human αSyn in mice. The titer of induced antibodies was further analyzed by enzyme-linked immunosorbent assay using the synthetic peptide αSyn_110–130_ as the target antigen (Fig 2C). The antibody titers were measured in the range of 3,200–25,600 folds of dilution with an average titer of 15,085 ± 3,870 in the post-Vac sera (n = 7). In contrast, antigen-specific antibodies were not found in the pre-Vac sera (n = 7). We next examined the specificity of induced antibodies in a competition assay (Fig 3). αSyn_110–130_ or a control peptide (Spike-binder) [38], together with pre- or post-Vac sera, were used to stain HEK293/pAAV-αSyn cells. αSyn_110–130_, but not Spike-binder, effectively reduced the green fluorescence signals of Alexa488-conjugated anti-mouse IgG polyclonal antibodies in a dose-dependent manner. No obvious green fluorescence signals were detected in the naive cells for all competition conditions. These data suggest that the Ct-αSyn complex vaccine induced active production of antibodies specifically targeting the human αSyn C_110–130_ terminal region.

### Overexpression of αSyn in the substantia nigra caused hypokinesia

**Fig 4:**
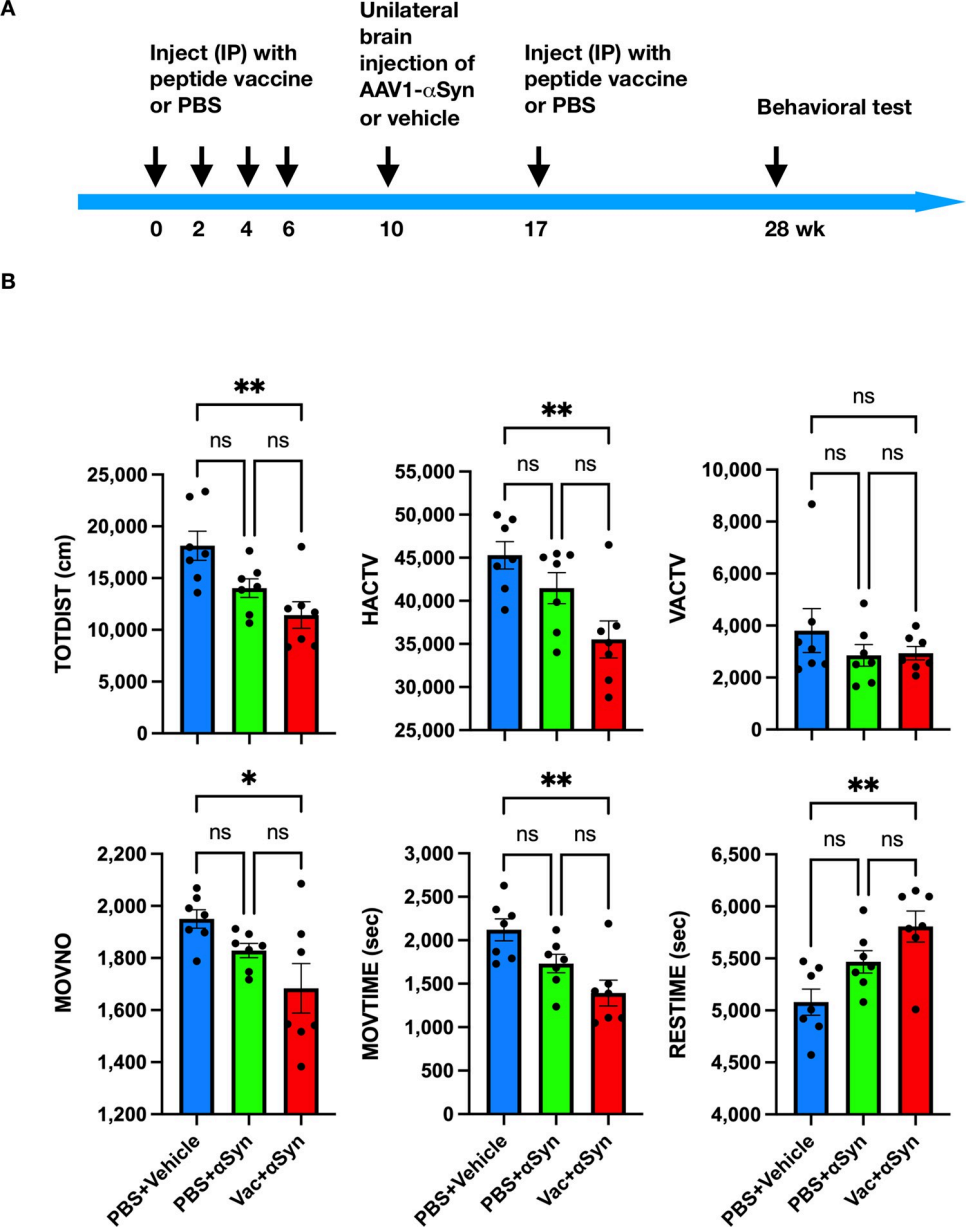
Ct-αSyn complex vaccine deteriorates motor function in mice overexpressing αSyn in the substantia nigra. (A) Experiment timeline. Adult mice were randomly divided into three groups and intraperitoneally injected with PBS (group 1: n = 7, blue, PBS + vehicle; group 2: n = 7, green, PBS + αSyn) or Ct-αSyn complex vaccine (group 3: n = 7, red, Vac + αSyn) on weeks 0, 2, 4, 6, and 17. On week 10, mice received a unilateral brain injection of AAV1-αSyn (groups 2 and 3) or vehicle (group 1) in the right substantia nigra. The behavioral test was performed in the activity chamber for 2 h on week 28. (B) Six parameters of locomotor activity were recorded, including total distance traveled (TOTDIST), horizontal activity (HACTV), vertical activity (VACTV), movement number (MOVNO), movement time (MOVTIME), and rest time (RESTIME). Data are expressed as mean ± SEM, and differences between groups were determined by ordinary ANOVA followed by Tukey’s multiple comparison tests (*p < 0.05, **p < 0.01, ns: not significant).

Adult mice (n = 14) were intraperitoneally injected with PBS on weeks 0, 2, 4, 6, and 17 to simulate vaccination. AAV1-αSyn (n = 7) or vehicle (n = 7) were stereotactically injected to the right substantia nigra on week 10. Locomotor activity was monitored on week 28 (see timeline in Fig 4A). AAV1-αSyn administration reduced total distance traveled (TOTDIST; 14,028 ± 900 vs. 18,125 ± 1,402; p = 0.0688), horizontal activity (HACTV; 41,469 ± 1,801 vs. 45,279 ± 1,587; p = 0.3388), vertical activity (VACTV; 2,851 ± 417 vs. 3,808 ± 844; p = 0.4686), movement numbers (MOVNO; 1,829 ± 27 vs. 1,950 ± 36; p = 0.3573), and movement time (MOVTIME; 1,732 ± 107 vs. 2,120 ± 126; p = 0.1113), while increased rest time (RESTIME; 5,468 ± 107 vs. 5,080 ± 126; p = 0.1113) (Fig 4B; group 2 vs. group 1). These results suggest that AAV-mediated overexpression of human αSyn in the substantia nigra induced hypokinesia in mice.

### Ct-αSyn complex vaccination aggravated αSyn-induced motor deficits in mice

Mice receiving the Ct-αSyn complex vaccine (n = 7; Vac + αSyn) or PBS (n = 7; PBS + αSyn) were injected with AAV1-αSyn in the right substantia nigra and then examined for locomotor behavior (see timeline in Fig 4A). Compared to the non-vaccinated control (group 2: PBS + αSyn), vaccinated mice (group 3: Vac + αSyn) exhibited a reduction in the total distance traveled (TOTDIST; 11,431 ± 1,279 vs. 14,023 ± 890; p = 0.3079), horizontal activity (HACTV; 35,508 ± 2,152 vs. 41,469 ± 1,801; p = 0.0872), movement numbers (MOVNO; 1,684 ± 95 vs. 1,829 ± 27; p = 0.2373), movement time (MOVTIME; 1,393 ± 149 vs. 1,732 ± 107; p = 0.1776), and an increase in the rest time (RESTIME; 5,807 ± 149 vs. 5,468 ± 107; p = 0.1776) and a marginal increase in the vertical activity (VACTV; 2,936 ± 260 vs. 2,851 ± 417; p = 0.9938) (Fig 4B; group 3 vs. group 2). When compared to the naive group (group 1: PBS + Vehicle), vaccinated mice (group 3: Vac + αSyn) exhibited a significant reduction in the total distance traveled (TOTDIST; 11,431 ± 1,279 vs. 18,125 ± 1,402; p = 0.0028), horizontal activity (HACTV; 35,508 ± 2,152 vs. 45,279 ± 1,587; p = 0.0043), movement numbers (MOVNO; 1,684 ± 95 vs. 1,950 ± 36; p = 0.0163), and movement time (MOVTIME; 1,393 ± 149 vs. 2,120 ± 126; p = 0.0023), and a marginal reduction in the vertical activity (VACTV; 2,936 ± 260 vs. 3,808 ± 844; p = 0.5305) and a significant increase in the rest time (RESTIME; 5,807 ± 149 vs. 5,080 ± 126; p = 0.0023) (Fig 4B; group 3 vs. group 1). These results suggest that Ct-αSyn complex vaccination exacerbated αSyn-induced motor deficits in mice.

### Ct-αSyn complex vaccination increased the accumulation of αSyn accompanied by neuroinflammation and neuronal damage

**Fig 5:**
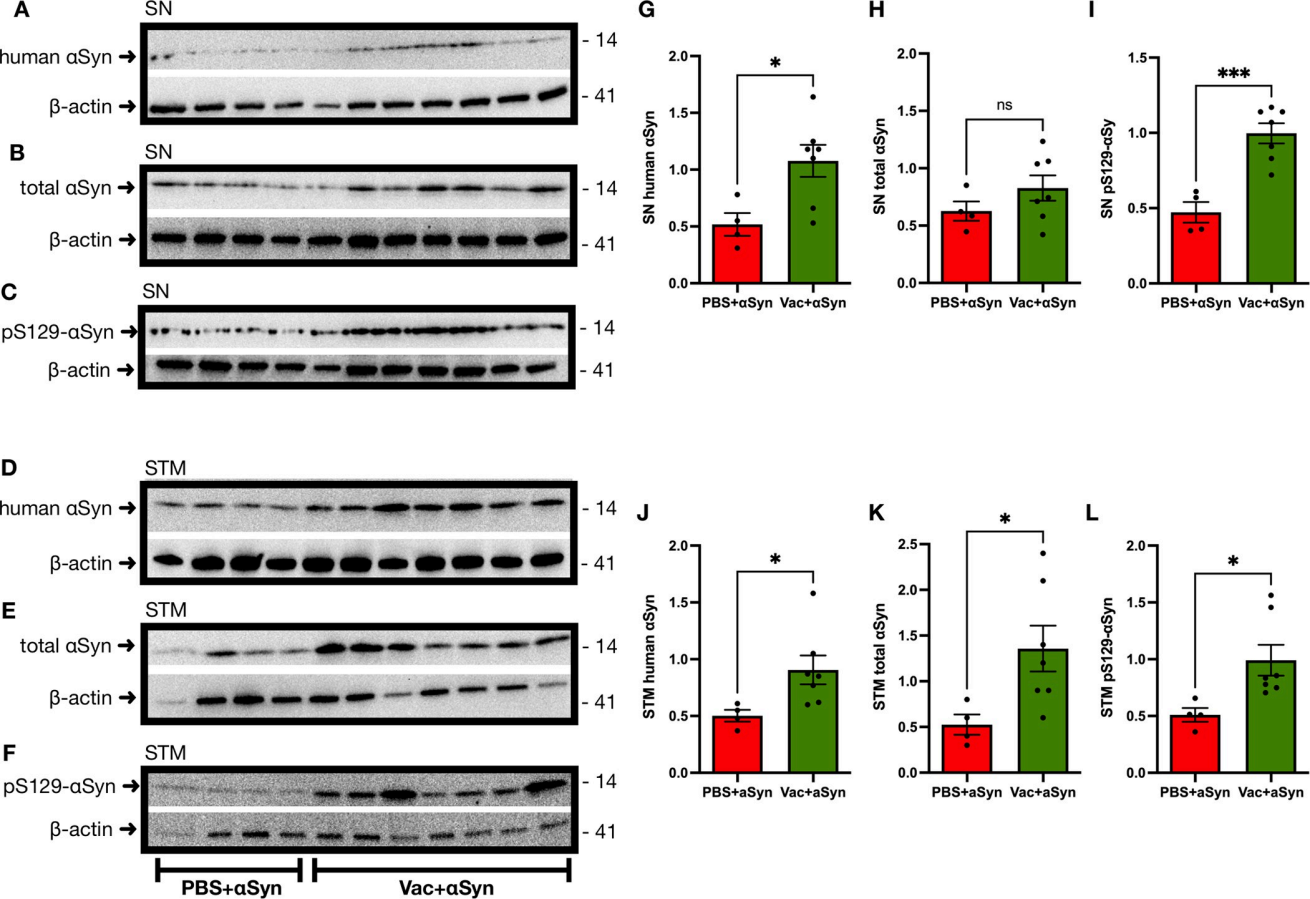
Ct-αSyn complex vaccine enhances the accumulation of αSyn and phosphorylated αSyn in the brain. Vaccinated mice (n = 7; Vac + αSyn) and non-vaccinated mice (n = 4; PBS + αSyn) were intracranially injected with the viral vector AAV1-αSyn in the right substantia nigra for overexpressing human αSyn. (A—F) Twenty weeks later, substantia nigra (SN) and striatum (STM) tissues of the right hemisphere brain were collected for Western blot analysis to probe human αSyn (by anti-V5 tag antibody), total αSyn (by BD clone-42 antibody), phosphorylated αSyn (pS129-αSyn), and β-actin. The molecular weight (kDa) and migration location of protein markers are indicated. (G—L) The levels of detected proteins were quantified by densitometric analysis and normalized to β-actin levels. Data are expressed as mean values ± SEM. Significant differences between groups are indicated (*p < 0.05, ***p < 0.001, ns: not significant; Student’s *t*-test).

**Fig 6:**
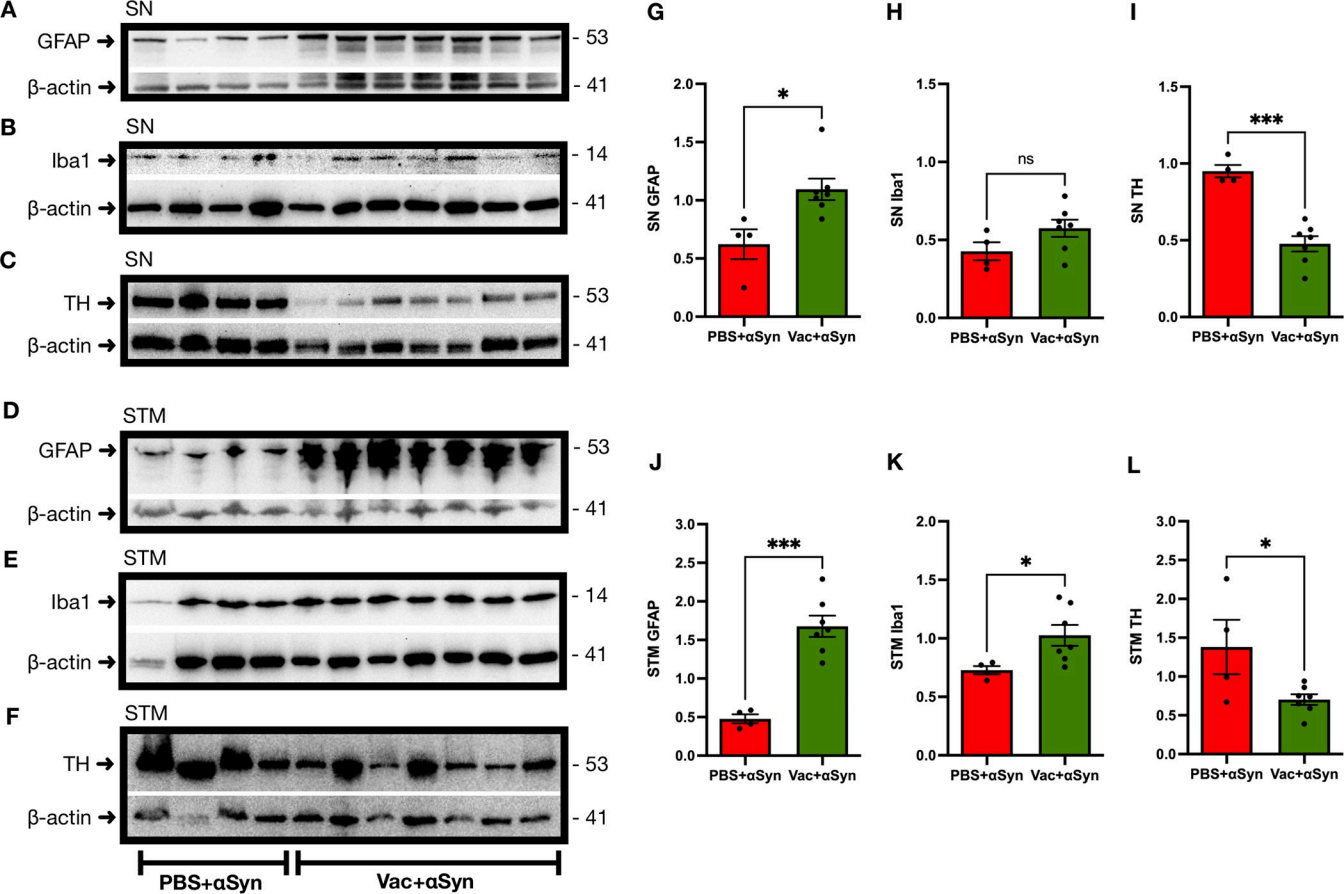
Ct-αSyn complex vaccine induces neuroinflammation and reduces TH levels. Vaccinated mice (n = 7; Vac + αSyn) and non-vaccinated mice (n = 4; PBS + αSyn) received an intracranial injection of the viral vector AAV1-αSyn in the right substantia nigra for overexpressing human αSyn. (A—F) Twenty weeks later, substantia nigra (SN) and striatum (STM) tissues of the right hemisphere brain were subjected to Western blot analysis to detect glial fibrillary acidic protein (GFAP), ionized calcium-binding adaptor molecule-1 (Iba1), tyrosine hydroxylase (TH), and β-actin. The molecular weight (kDa) and migration location of protein markers are indicated. (G—L) The protein levels of GFAP, Iba1, and TH were quantified by densitometric analysis and normalized to β-actin levels. Data are expressed as mean values ± SEM. Significant differences between groups are indicated (*p < 0.05, ***p < 0.001, ns: not significant; Student’s *t*-test).

**Fig 7:**
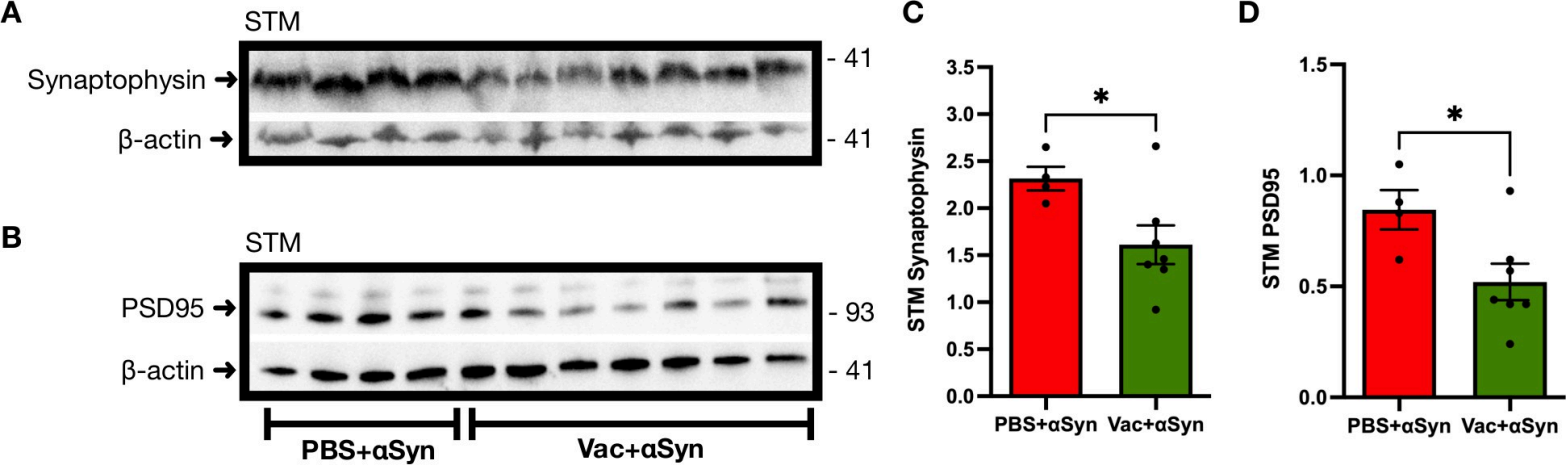
Ct-αSyn complex vaccine downregulates synaptic markers. Vaccinated mice (n = 7; Vac + αSyn) and non-vaccinated mice (n = 4; PBS + αSyn) were administered with the viral vector AAV1-αSyn in the right substantia nigra for overexpressing human αSyn. (A, B) Twenty weeks later, striatum (STM) tissues of the right hemisphere brain were examined by western blot analysis to probe synaptophysin, postsynaptic density protein-95 (PSD95), and β-actin. The molecular weight (kDa) and migration location of protein markers are indicated. (C, D) The protein levels of synaptophysin and PSD95 were measured and normalized to β-actin by densitometric analysis. Data are expressed as mean values ± SEM, and significant differences between groups are indicated (*p < 0.05; Student’s *t*-test).

On week 20, after intracranial injection of AAV1-αSyn, the brain tissues of vaccinated mice (n = 7; Vac + αSyn) and non-vaccinated mice (n = 4; PBS + αSyn) were collected for Western blotting analysis to probe human αSyn, total αSyn, phosphorylated αSyn (pS129-αSyn), GFAP, TH, PSD95, synaptophysin, and β-actin. The protein levels of the detected targets were quantified by densitometric analysis and normalized to β-actin levels. The levels of human αSyn (substantia nigra: 1.08 ± 0.14 vs. 0.52 ± 0.10, p = 0.0232, Fig 5A and 5G; striatum: 0.91 ± 0.13 vs. 0.5 ± 0.05, p = 0.0471, Fig 5D and 5J), total αSyn (striatum: 1.36 ± 0.25 vs. 0.53 ± 0.11, p = 0.0413, Fig 5E and 5K), and pS129-αSyn (substantia nigra: 1.00 ± 0.07 vs. 0.47 ± 0.07, p = 0.0007, Fig 5C and 5I; striatum: 0.99 ± 0.14 vs. 0.51 ± 0.06, p = 0.0311, Fig 5F and 5L) were significantly higher in the vaccinated mice than in the non-vaccinated mice; total αSyn in the substantia nigra (0.83 ± 0.11 vs. 0.63 ± 0.08, p = 0.2443, Fig 5B and 5H) was also higher but not significant. These results indicate that Ct-αSyn complex vaccination increased the accumulation of αSyn. Compared to non-vaccinated controls, vaccinated mice showed greater neuroinflammation, observed as a significant increase in GFAP (substantia nigra: 1.09 ± 0.09 vs. 0.62 ± 0.13, p = 0.0142, Fig 6A and 6G; striatum: 1.68 ± 0.14 vs. 0.48 ± 0.06, p = 0.0001, Fig 6D and 6J), and Iba1 (striatum: 1.03 ± 0.09 vs. 0.73 ± 0.04, p = 0.0395, Fig 6E and 6K); Iba1 was also increased in the substantia nigra (0.58 ± 0.05 vs. 0.43 ± 0.06, p = 0.1182, Fig 6B and 6H) but without significance. The dopaminergic neuron marker TH (substantia nigra: 0.48 ± 0.05 vs. 0.95 ± 0.04, p = 0.0001, Fig 6C and 6I; striatum: 0.70 ± 0.07 vs. 1.38 ± 0.35, p = 0.0337, Fig 6F, 6L) was detected at a significantly lower level in vaccinated mice than in non-vaccinated controls. In the striatum, the presynaptic marker synaptophysin (1.61 ± 0.21 vs. 2.32 ± 0.13, p = 0.0398, Fig 7A and 7C) and postsynaptic marker PSD95 (0.52 ± 0.08 vs. 0.845 ± 0.09, p = 0.0325, Fig 7B and 7D) were significantly down-regulated in vaccinated mice. The reduction of these neuronal markers suggests that Ct-αSyn complex vaccination induced synaptic loss and degeneration of dopaminergic neurons.

### Ct-αSyn complex vaccination elicited mild inflammation but did not increase tissue damage or αSyn accumulation in the periphery

**Fig 8:**
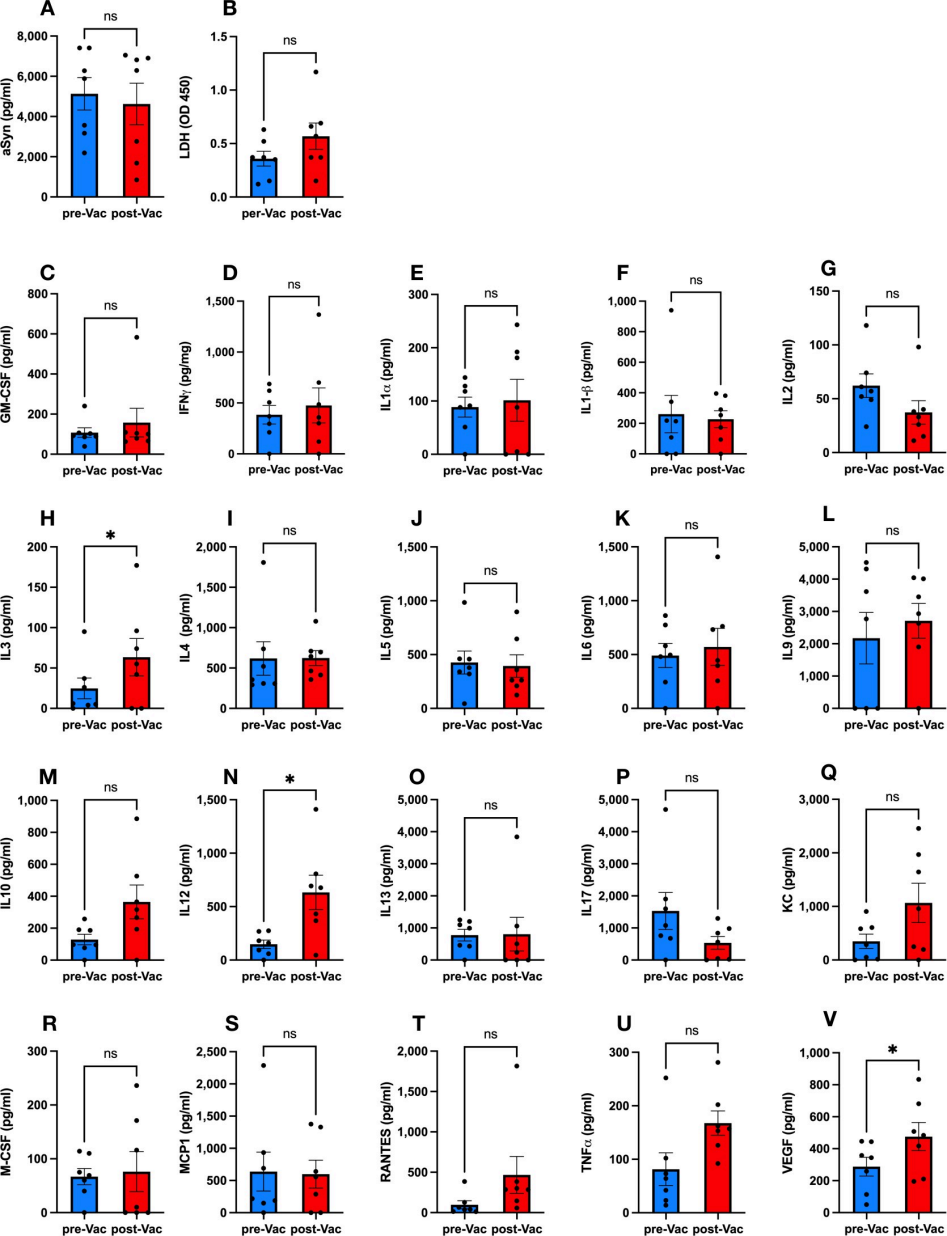
Peripheral levels of αSyn, LDH, and cytokines in the mice vaccinated with the Ct-αSyn complex. Mice were intraperitoneally injected with the Ct-αSyn complex (n = 7) on weeks 0, 2, 4, 6, and 17. On week 10, mice received a unilateral brain injection of AAV1-αSyn in the right substantia nigra and were subjected to behavioral tests on week 28. Peripheral blood sera were collected on days -2 (pre-Vac; 2 days before the first vaccination) and 60 (post-Vac: 18 days after the fourth vaccination) for the measurement of αSyn (A), lactate dehydrogenase (B) and 20 inflammation-related cytokines (C—V). Data were expressed as mean concentrations (pg/ml or OD_450_) ± SEM, and differences between groups were determined by the paired, two-tailed Student’s t-test (*p < 0.05, ns: not significant).

To investigate whether the administration of the Ct-αSyn complex can cause any physiological toxicity, we collected serum samples before (pre-Vac) and after (post-Vac) vaccination for the measurement of αSyn, lactate dehydrogenase (LDH; a marker for tissue damage), and 20 inflammation-related cytokines. In the post-Vac sera, αSyn (4622 ± 1036 vs. 5130 ± 805 pg/ml, p = 0.7241, Fig 8A) and LDH (0.57 ± 0.12 vs. 0.36 ± 0.07 OD_450_, p = 0.1315, Fig 8B) levels were similar to those in the pre-Vac sera. The levels of IL3 (63.43 ± 23.22 vs. 24.71 ± 12.79 pg/ml, p = 0.0335, Fig 8H), IL12 (633.3 ± 161.4 vs. 147.7 ± 39.14 pg/ml, p = 0.0346, Fig 8N), and VEGF (475.6 ± 87.9 vs. 287.9 ± 58.78 pg/ml, p = 0.046, Fig 8V) were significantly higher in the post-Vac sera than in the pre-Vac sera. The levels of IL10 (364.6 ± 105.9 vs. 129 ± 32.78 pg/ml, p = 0.1038, Fig 8M), KC (1067 ± 366.9 vs. 349.7 ± 134.4 pg/ml, p = 0.0684, Fig 8Q), RANTES (467.4 ± 228.5 vs. 97.43 ± 50.2 pg/ml, p = 0.1639, Fig 8T), and TNFα (167.6 ± 22.81 vs. 81.43 ± 30.63 pg/ml, p = 0.0725, Fig 8U) were over 2-fold higher in the post-Vac sera than in the pre-Vac sera, but without significance. The level of IL17 (535.6 ± 198.6 vs. 1529 ± 577.8 pg/ml, p = 0.2102, Fig 8P) was lower in the post-Vac sera than in the pre-Vac sera, but the difference was not significant. The levels of GM-CSF (157.7 ± 71.22 vs. 107.9 ± 23.56 pg/ml, p = 0.5522, Fig 8C), IFNγ (476.1 ± 172.1 vs. 384.1 ± 90.92 pg/ml, p = 0.7154, Fig 8D), IL1α (12.71 ± 54.45 vs. 88.57 ± 17.74 pg/ml, p = 0.8231, Fig 8E), IL1β (226.7 ± 55.37 vs. 260.1 ± 122.6 pg/ml, p = 0.8351, Fig 8F), IL2 (37.29 ± 10.94 vs. 62.14 ± 10.93 pg/ml, p = 0.2202, Fig 8G), IL4 (623.4 ± 92.71 vs. 618.7 ± 207.3 pg/ml, p = 0.9857, Fig 8I), IL5 (394 ± 104.6 vs. 426.4 ± 106.9 pg/ml, p = 0.8338, Fig 8J), IL6 (571.1 ± 171.4 vs. 491 ± 112.1 pg/ml, p = 0.7175, Fig 8K), IL9 (2710 ± 536.1 vs. 2173 ± 796.7 pg/ml, p = 0.6586, Fig 8L), IL13 (806.9 ± 526.3 vs. 776.7 ± 181.1 pg/ml, p = 0.9534, Fig 8O), M-CSF (76.14 ± 37.13 vs. 66.86 ± 14.95 pg/ml, p = 0.8268, Fig 8R), and MCP1 (598.9 ± 215.9 vs. 638 ± 302.1 pg/ml, p = 0.9248, Fig 8S) were similar between post-Vac and pre-Vac sera. These results suggest that administrating the Ct-αSyn complex induced a mild inflammatory response but enhanced neither tissue damage nor αSyn accumulation in the periphery.

## Discussion

This study aimed to investigate the production of antibodies, neurotoxicity, and motor function in αSyn-expressing animals receiving Ct-αSyn complex vaccination. Our data showed that the Ct-αSyn complex was able to elicit antibodies to recognize the C-terminal of αSyn. However, immunization with this Ct-αSyn complex increased αSyn accumulation and neuroinflammation accompanied by dopaminergic degeneration and synaptic damage in the brain. In addition, motor deficits were exacerbated in the vaccinated mice. Our data suggest that vaccination with the C-terminal peptide did not provide further protection in animals overexpressing αSyn. The application of the αSyn C-terminal vaccine for PD should be exercised with caution.

Antigen-specific B cells need to receive two major inputs for antibody production: (i) the crosslink of B cell surface immunoglobulin (Ig) and (ii) cytokine stimulation from activated helper T cells [26,40]. These two signals can be activated by the Ct-αSyn complex. For example, the Ct-αSyn complex contains an arginine-rich motif, which allows its binding to cell surfaces to present multiple copies of αSyn_110–130_ to B cells to induce surface Ig crosslink [33]. The helper T cell response induced by the PADRE epitope is strong enough to activate αSyn_110–130_-specific B cells without the requirement of clustering surface Ig receptors by multivalent αSyn_110–130_ [32]. In contrast to the traditional liner peptides with insufficient antibody production [41,42], the Ct-αSyn complex induced antibody production to titers in the range of 3,200–25,600 folds of dilution. Our data support that immunization with the Ct-αSyn complex effectively triggered the synthesis of αSyn-specific antibodies. The sequence design of the fusion peptides may be of great potential for the development of other peptide-based vaccines targeting the epitopes of interest.

Theoretically, antigen-presenting cells, such as dendritic cells and macrophages, can capture the complex peptide PADRE-Tat-Ct-αSyn via endocytosis and degrade it into peptides and amino acids by resident proteases in the early endosome and MHC class II compartment (MIIC). In the MIIC, free PADRE peptides can fit in the peptide-binding groove of MHC-II proteins and are then transported to the cell surface as peptide/MHC-II complexes to stimulate CD4+ helper T cells [43]. The peptide complex PADRE-Tat-Ct-αSyn was designed to induce antibody responses against the Ct-αSyn peptide. In the extracellular microenvironment, it is possible that if the PADRE portion binds to its receptors (MHC-II proteins), the Tat portion cannot attach to the cell surface, and the converse would also be true. Therefore, including the Tat sequence in this peptide vaccine might not be necessary for developing antibodies. However, the Tat sequence is a cell-penetrating peptide to facilitate the cellular uptake of extracellular molecules associated with it [44,45]. Therefore, it can enhance the delivery of PADRE-Tat-Ct-αSyn into antigen-presenting cells, increasing PADRE presentation and activation of CD4+ helper T cells for antibody production by B cells. Due to the presence of the Tat sequence, the complex peptide PADRE-Tat-Ct-αSyn could be superior to PADRE-Ct-αSyn in terms of efficacy in developing antibodies against the Ct-αSyn peptide.

The present study showed that active immunization with Ct-αSyn complex further deteriorated motor function in αSyn mice. This adverse effect could be attributed to the synaptic damage and degeneration of dopaminergic neurons in the nigrostriatal pathway, as the protein levels of synaptic markers and TH were significantly reduced while αSyn expression was further enhanced in vaccinated mice. However, a motor test performed before the AAV-αSyn injection is required to examine whether the vaccination can cause any baseline shifts in the motor function. As the nigral TH^+^ cell count and striatal axonal density were not examined by immunohistochemistry staining, we should consider the suggestion of neurodegeneration in the vaccinated animals with caution. On the other hand, an increased level of phosphorylation of αSyn at serine129 (pS129 αSyn) was found in vaccinated mice. Although the role of pS129 αSyn in regulating αSyn-associated neurotoxicity remains debated, the accumulation of pS129 αSyn is considered a promising marker for PD and synucleinopathies [46–48]. Furthermore, astrogliosis occurred in vaccinated mice, as increased expression of the astroglial marker GFAP was detected. During the process of astrogliosis, astrocytes can release proinflammatory cytokines under pathological conditions such as aberrant accumulation of αSyn [15,49]. It is possible that neuroinflammation also plays a role in neuronal damage in vaccinated mice, supported by the elevation of GFAP and microglia marker Iba1. Furthermore, as an increased intensity of inflammatory markers (GFAP, Iba1) was identified in the brain of vaccinated mice, the Ct-αSyn complex could potentially over-activate inflammation in the brain and lead to the worsening of αSyn pathology and motor symptoms. Although administrating the Ct-αSyn complex elicited mild inflammation in the periphery, the increase of tissue damage and αSyn accumulation was not found in the sera collected after Ct-αSyn vaccination. However, we cannot rule out that vaccination with the Ct-αSyn complex before AAV-mediated overexpression of αSyn might generate a robust immune response against AAV injection in the brain and thus lead to neuronal inflammation and degeneration. Therefore, whether vaccination with the Ct-αSyn complex after AAV injection can avoid inducing inflammation in the brain requires further investigation.

Of note, αSyn levels were measured from the soluble fraction of brain tissue lysates by Western blot analysis, but whether the increase in the soluble fraction is caused by the decrease in the insoluble fraction needs to be evaluated. In the vaccinated animals, whether the decrease of TH protein is related to the loss of TH+ dopaminergic neurons, and whether the elevation of GFAP and Iba1 is due to increased reactivity or cell counts require further investigation by immunohistochemistry analysis.

The mechanism of αSyn accumulation induced by the vaccination of the Ct-αSyn complex is unclear, but the following facts could partly explain it. αSyn is prevalently modified by phosphorylation at serine residues (S87 and S129) and tyrosine residues (Y125, Y133, and Y135), ubiquitination at lysine residues (K6, K10, and K12), as well as nitration at tyrosine residues (Y39, Y125, Y133, and Y136), which are implicated in the process of αSyn aggregation and degradation [50–54]. Phosphorylation of S129 or nitration of Y125, Y133, and Y136 has been reported to increase αSyn accumulation [46,50]. Our peptide vaccine promoted the phosphorylation of S129, as increased levels of pS129 αSyn were found in the substantia nigra and striatum of vaccinated mice. On the other hand, the interactions between the C-terminal and N-terminal or central NAC region were indicated to stabilize the native structure of αSyn and thus prevent αSyn aggregation [55]. Our peptide vaccine induces antibodies to selectively target the C-terminal region of αSyn, which might disrupt these intramolecular interactions and promote αSyn accumulation. However, more studies are needed to examine these possibilities.

In contrast to our findings, two other reports by Masliah et al. and Mandler et al. demonstrated that immunization with recombinant full-length human αSyn [17] or a carrier-conjugated peptide vaccine [18] reduced αSyn accumulation in transgenic mice expressing human αSyn. The discrepancy may be attributed to multiple factors. (A) Timing of vaccination. In Masliah’s and Mandler’s studies, αSyn-specific antibodies were induced after αSyn was fully expressed in transgenic mice [17,18]. In our study, the Ct-αSyn complex was introduced prior to the expression of αSyn, a more clinically relevant approach. During vaccine stimulation, endogenous human αSyn might play a role in modulating antibody affinity in favor of the clearance of human αSyn aggregates in transgenic mice. (B) Masliah’s vaccine-induced antibodies recognize a broader region, including amino acid residues 85–99, 109–123, 112–126, and 126–138 of human αSyn [17]. In our study, Ct-αSyn complex-induced antibodies were designed selectively for the C-terminal residues 110–130 of human αSyn. It is possible that multiple targets to the αSyn C-terminal are needed to slow down the formation and aggregation of αSyn. (C) The vaccine peptide used in Mandler’s study contained an 8-amino acid non-native sequence simulating the structure of the human αSyn C-terminal (110–130 residues). This short peptide was not recognized by MHC-I and MHC-II to activate αSyn-specific T cells [18]. Our vaccine contained a 21-amino acid peptide (110–130 residues of human αSyn). The longer peptide may activate T cells to attack αSyn-expressing neurons and lead to dopaminergic degeneration in αSyn mice.

## Conclusions

In conclusion, we demonstrate that the Ct-αSyn complex effectively promoted the production of epitope-specific antibodies in vivo. However, vaccination did not improve behavior or reduce dopaminergic degeneration in animals overexpressing αSyn. The application of αSyn C-terminal peptide vaccine for PD should be exercised carefully.

## Supporting information

S1 Fig Raw images of western blot results. (PDF)
