# Supplementary material for: Peptide immunization against the C-terminal of alpha-synuclein reduces locomotor activity in mice overexpressing alpha-synuclein
Source: PLoS One. 2023 Sep 21;18(9):e0291927. doi: 10.1371/journal.pone.0291927 (PMC10513202; doi:10.1371/journal.pone.0291927)
Supplement: S1 Fig — (PDF) [file pone.0291927.s001.pdf]

Figure 2B

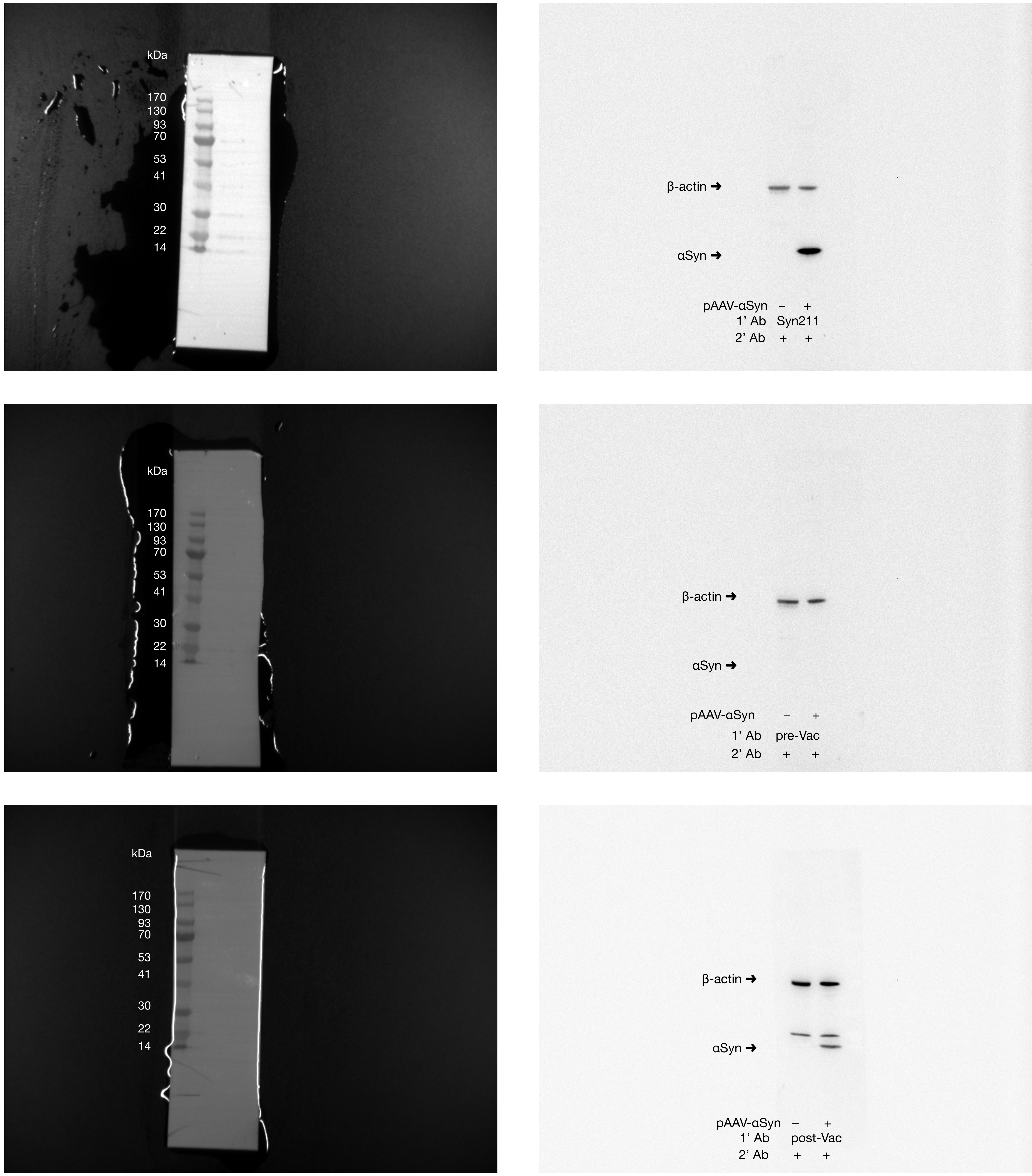
kDa
170
130
93
70
53
41
30
22
14
β-actin ➜
αSyn ➜
pAAV-αSyn – +
1’ Ab Syn211
2’ Ab + +
kDa
170
130
93
70
53
41
30
22
14
β-actin ➜
αSyn ➜
pAAV-αSyn – +
1’ Ab pre-Vac
2’ Ab + +
kDa
170
130
93
70
53
41
30
22
14
β-actin ➜
αSyn ➜
pAAV-αSyn – +
1’ Ab post-Vac
2’ Ab + +

Figure 5A: human αSyn

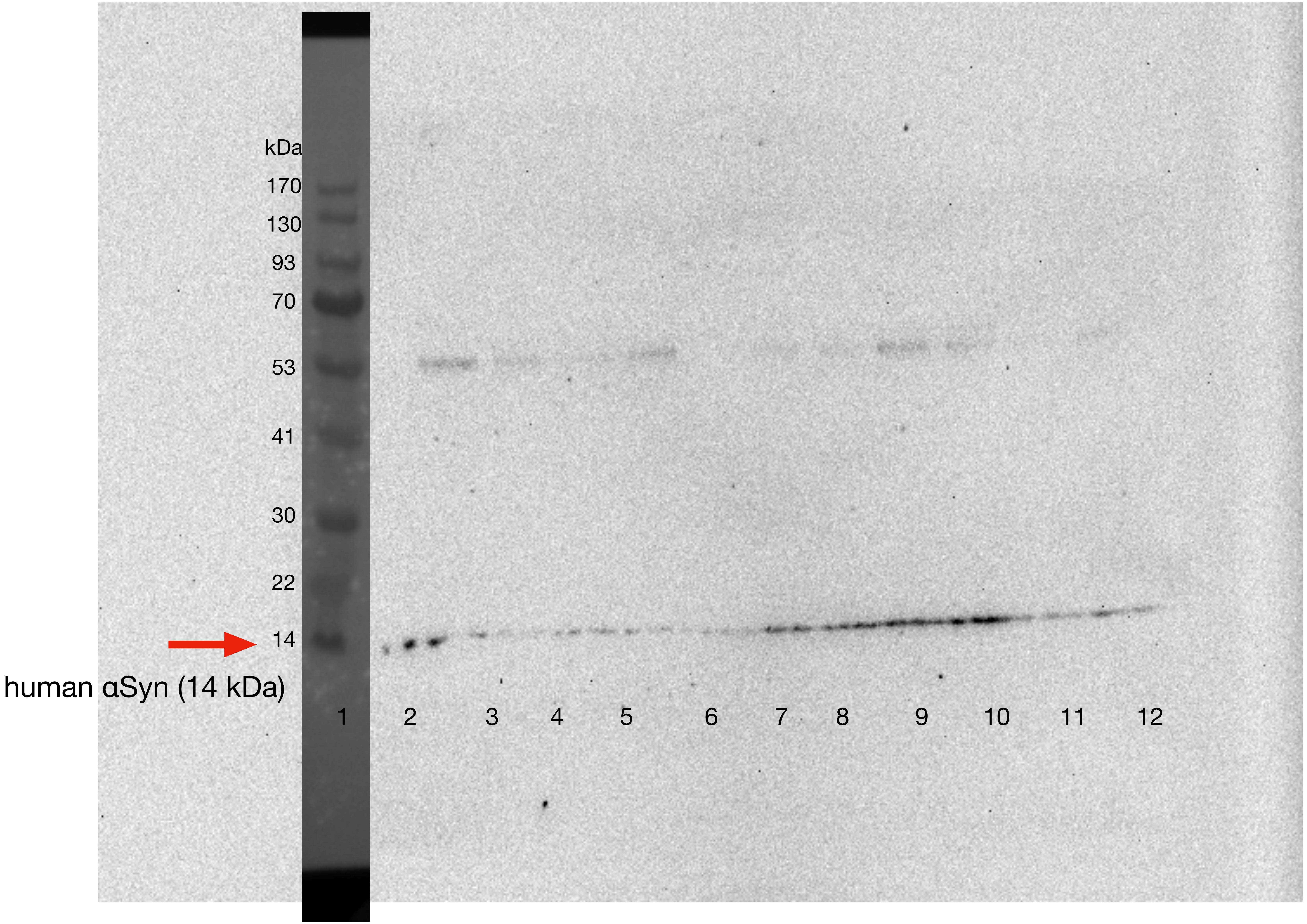

**1**	Marker
**2**	PBS+aSyn 1
**3**	PBS+aSyn 2
**4**	PBS+aSyn 3
**5**	PBS+aSyn 4
**6**	Vac+aSyn 1
**7**	Vac+aSyn 2
**8**	Vac+aSyn 3
**9**	Vac+aSyn 4
**10**	Vac+aSyn 5
**11**	Vac+aSyn 6
**12**	Vac+aSyn 7

Figure 5A: β-actin

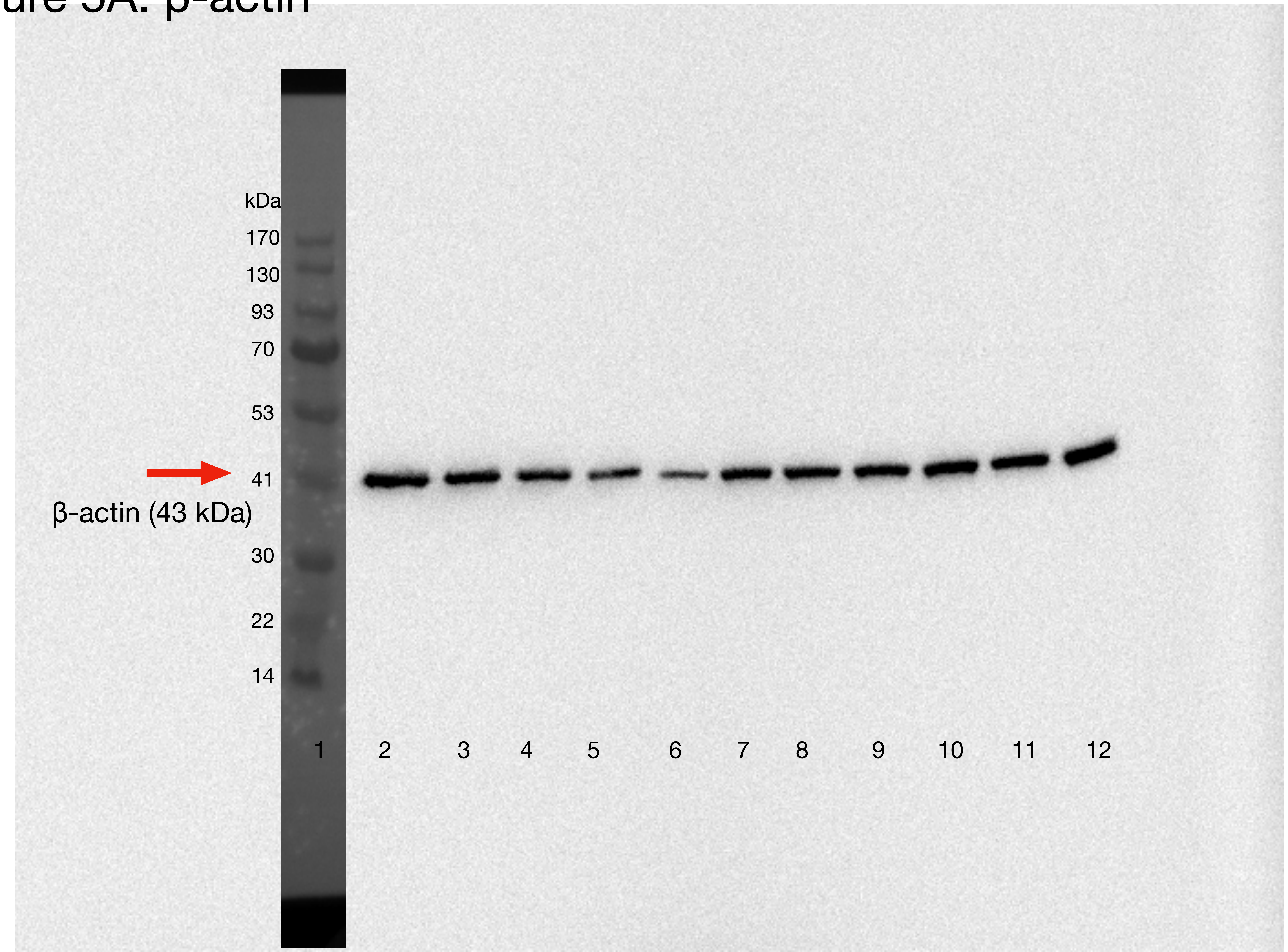

**1**	Marker
**2**	PBS+aSyn 1
**3**	PBS+aSyn 2
**4**	PBS+aSyn 3
**5**	PBS+aSyn 4
**6**	Vac+aSyn 1
**7**	Vac+aSyn 2
**8**	Vac+aSyn 3
**9**	Vac+aSyn 4
**10**	Vac+aSyn 5
**11**	Vac+aSyn 6
**12**	Vac+aSyn 7

Figure 5B: total αSyn

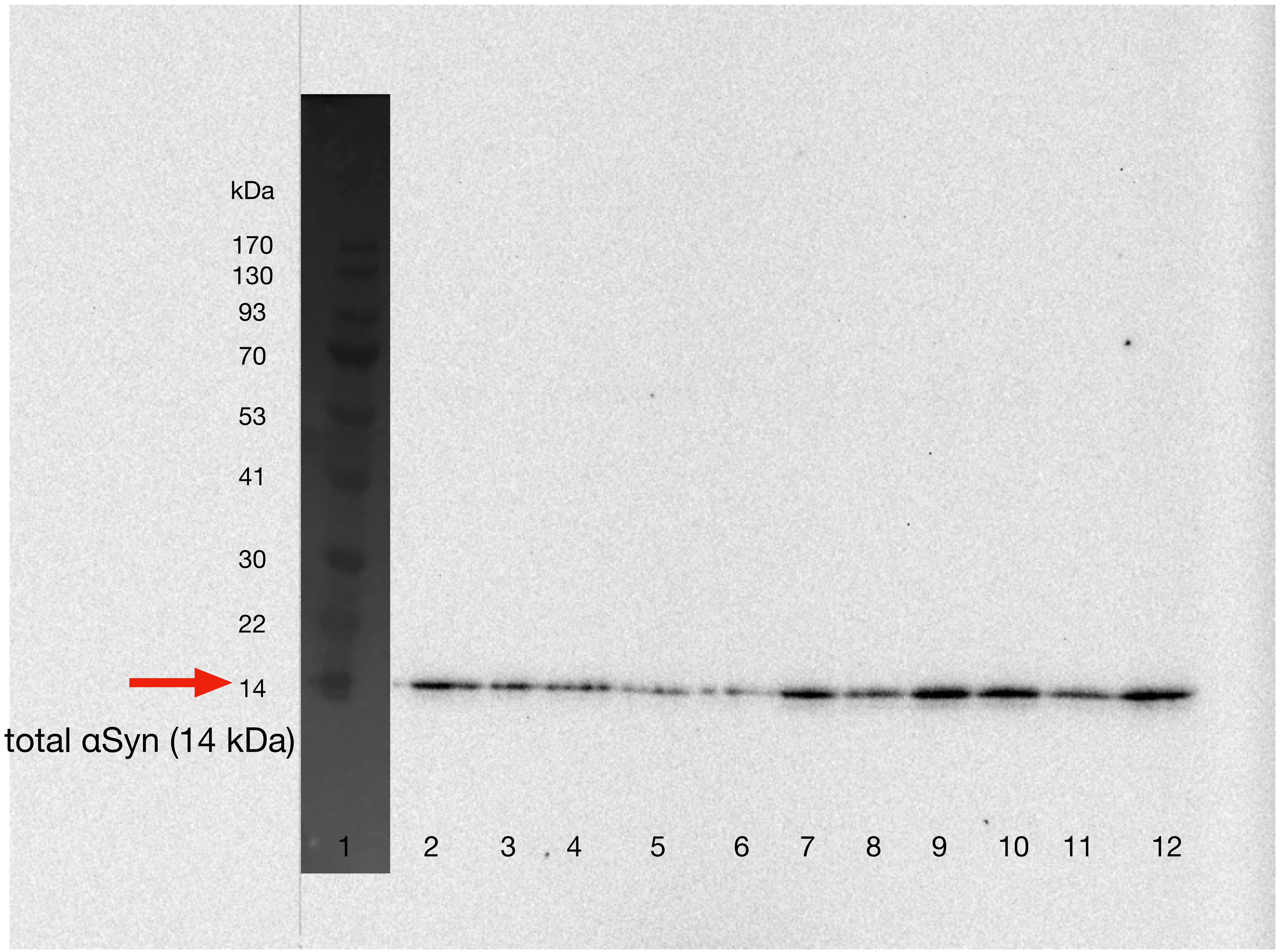

1	Marker
2	PBS+aSyn 1
3	PBS+aSyn 2
4	PBS+aSyn 3
5	PBS+aSyn 4
6	Vac+aSyn 1
7	Vac+aSyn 2
8	Vac+aSyn 3
9	Vac+aSyn 4
10	Vac+aSyn 5
11	Vac+aSyn 6
12	Vac+aSyn 7

Figure 5B: β-actin

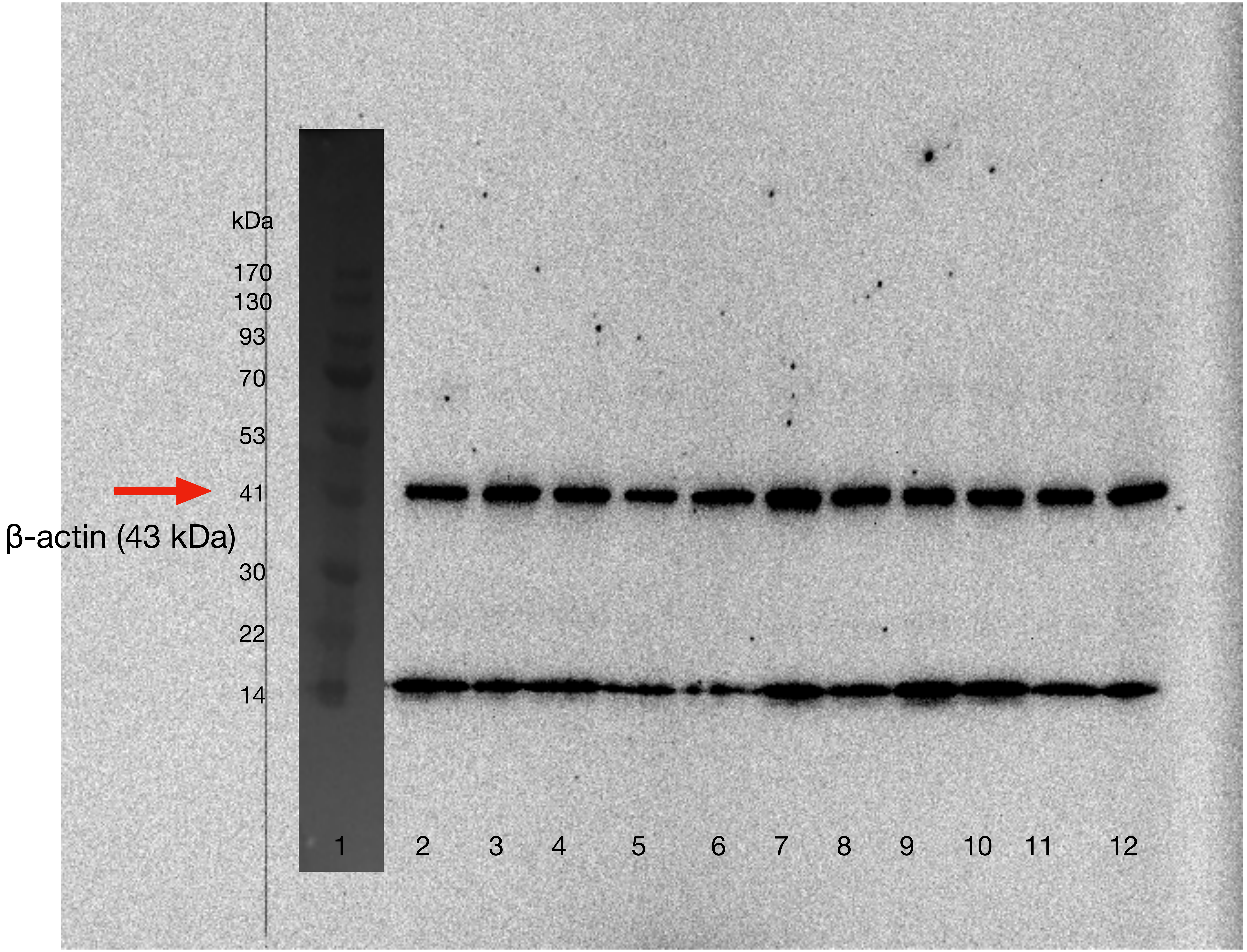

**1**	Marker
**2**	PBS+aSyn 1
**3**	PBS+aSyn 2
**4**	PBS+aSyn 3
**5**	PBS+aSyn 4
**6**	Vac+aSyn 1
**7**	Vac+aSyn 2
**8**	Vac+aSyn 3
**9**	Vac+aSyn 4
**10**	Vac+aSyn 5
**11**	Vac+aSyn 6
**12**	Vac+aSyn 7

Figure 5C: pS129-αSyn

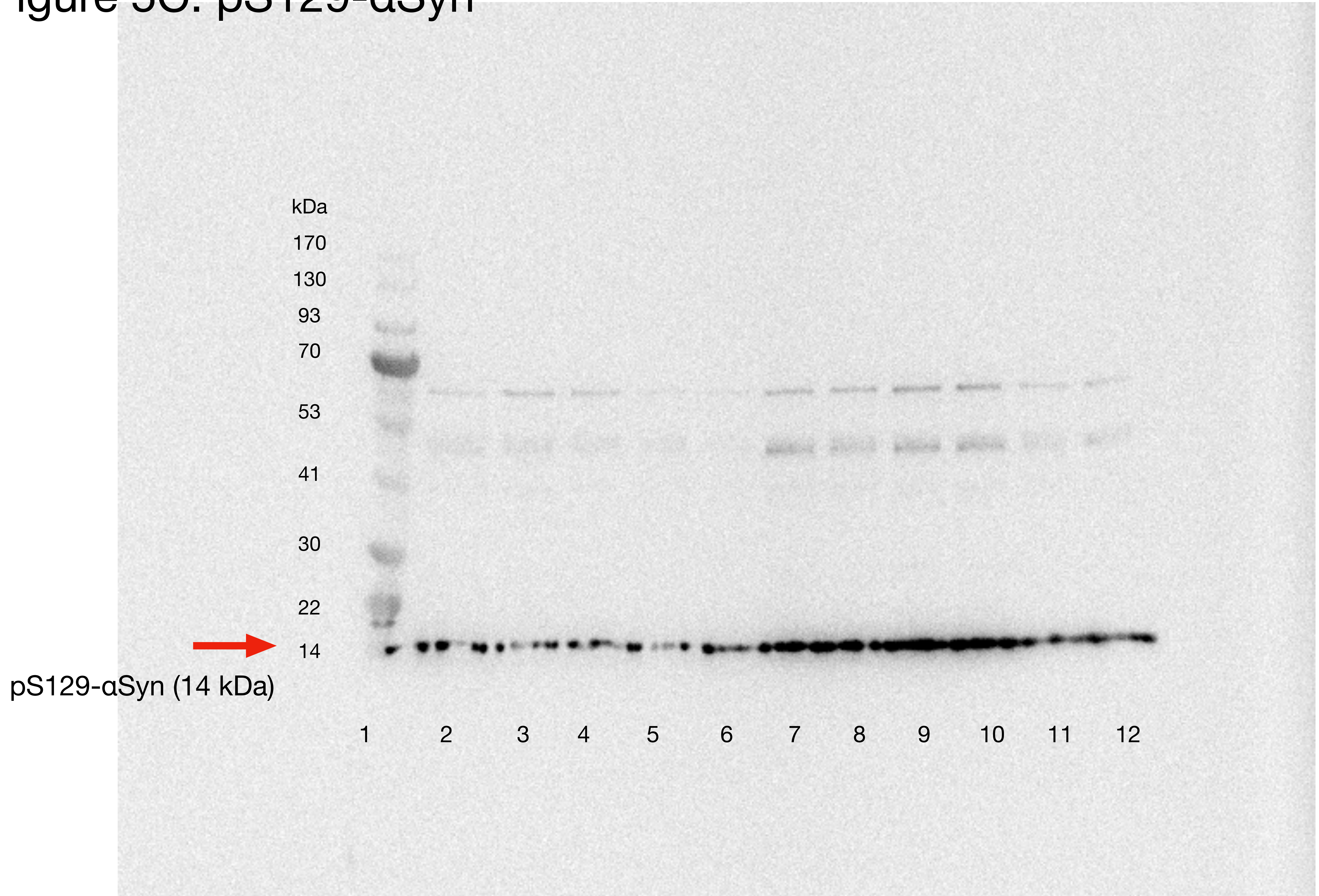

**1**	Marker
**2**	PBS+aSyn 1
**3**	PBS+aSyn 2
**4**	PBS+aSyn 3
**5**	PBS+aSyn 4
**6**	Vac+aSyn 1
**7**	Vac+aSyn 2
**8**	Vac+aSyn 3
**9**	Vac+aSyn 4
**10**	Vac+aSyn 5
**11**	Vac+aSyn 6
**12**	Vac+aSyn 7

Figure 5C: β-actin

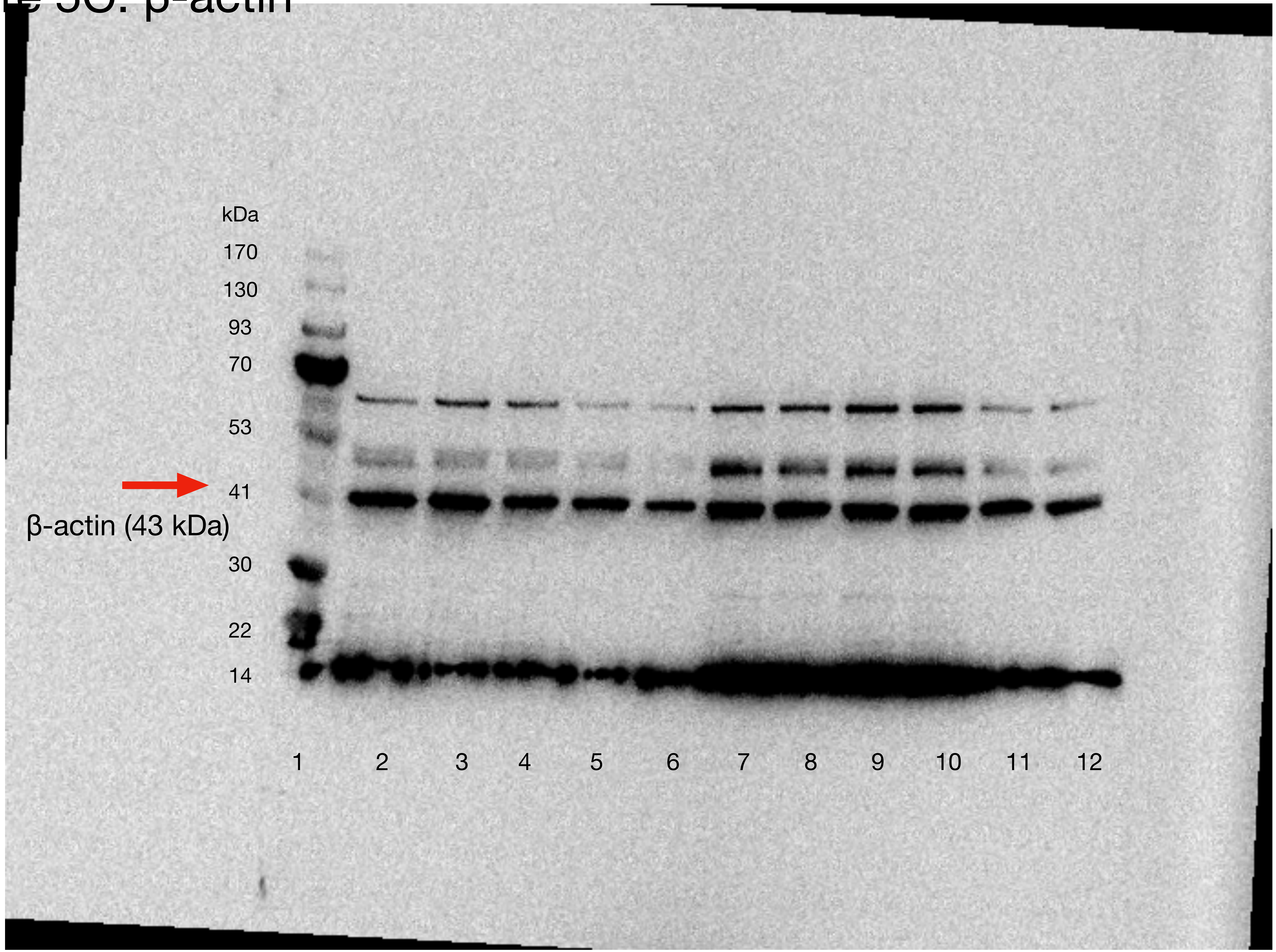

**1**	Marker
**2**	PBS+aSyn 1
**3**	PBS+aSyn 2
**4**	PBS+aSyn 3
**5**	PBS+aSyn 4
**6**	Vac+aSyn 1
**7**	Vac+aSyn 2
**8**	Vac+aSyn 3
**9**	Vac+aSyn 4
**10**	Vac+aSyn 5
**11**	Vac+aSyn 6
**12**	Vac+aSyn 7

Figure 5D: human αSyn

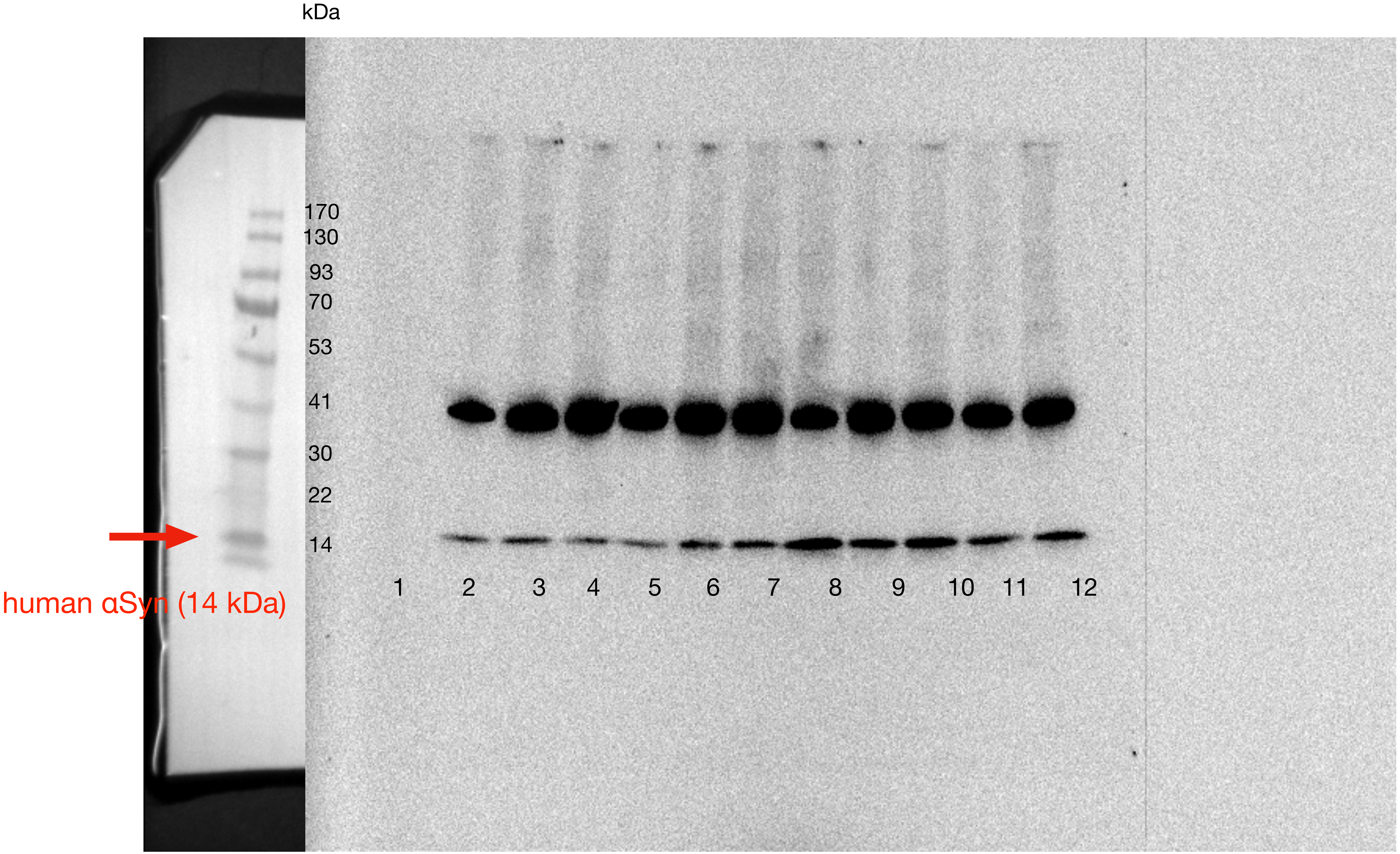

**1**	Marker
**2**	PBS+aSyn 1
**3**	PBS+aSyn 2
**4**	PBS+aSyn 3
**5**	PBS+aSyn 4
**6**	Vac+aSyn 1
**7**	Vac+aSyn 2
**8**	Vac+aSyn 3
**9**	Vac+aSyn 4
**10**	Vac+aSyn 5
**11**	Vac+aSyn 6
**12**	Vac+aSyn 7

Figure 5D: β-actin

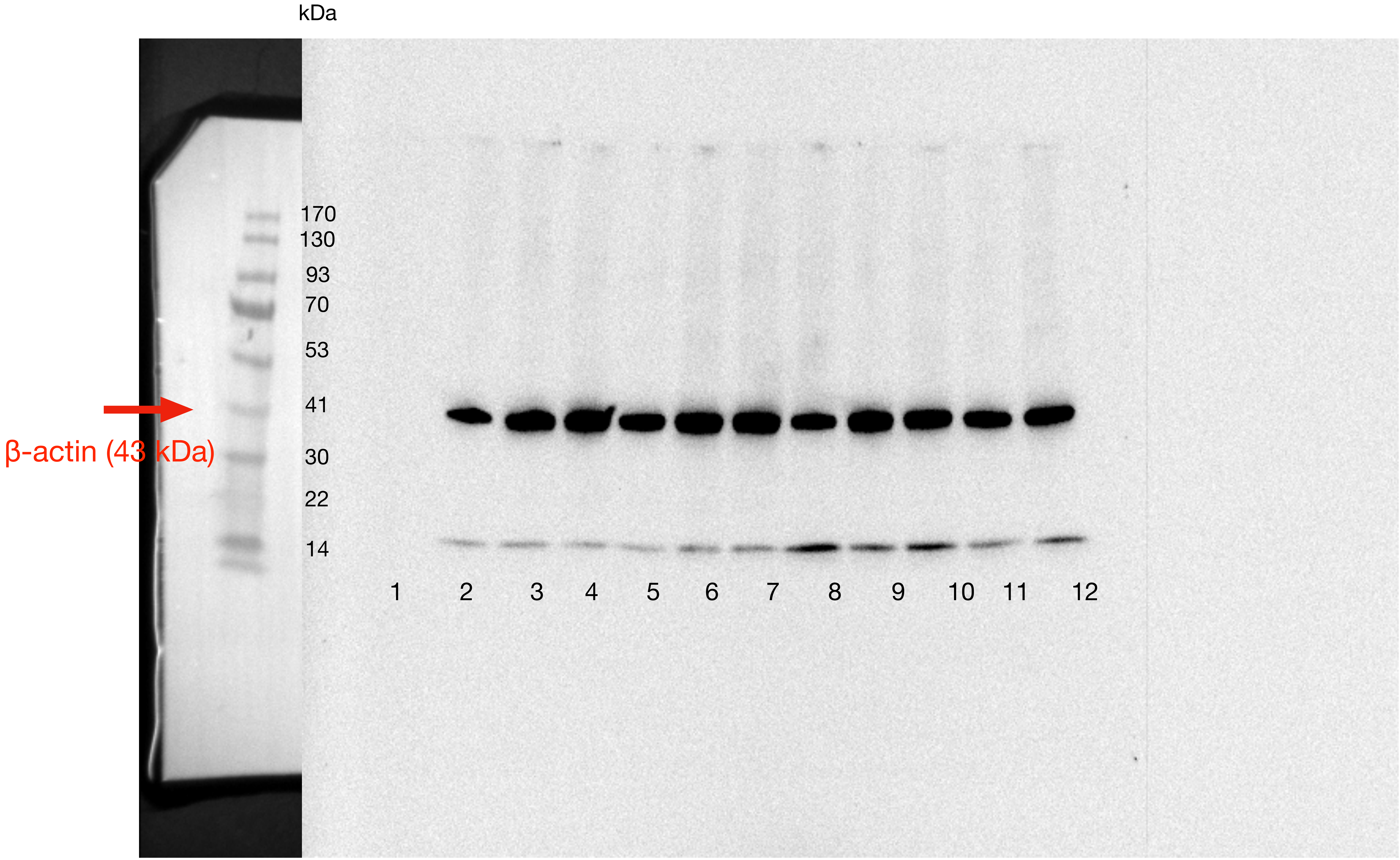

**1**	Marker
**2**	PBS+aSyn 1
**3**	PBS+aSyn 2
**4**	PBS+aSyn 3
**5**	PBS+aSyn 4
**6**	Vac+aSyn 1
**7**	Vac+aSyn 2
**8**	Vac+aSyn 3
**9**	Vac+aSyn 4
**10**	Vac+aSyn 5
**11**	Vac+aSyn 6
**12**	Vac+aSyn 7

Figure 5E: total αSyn

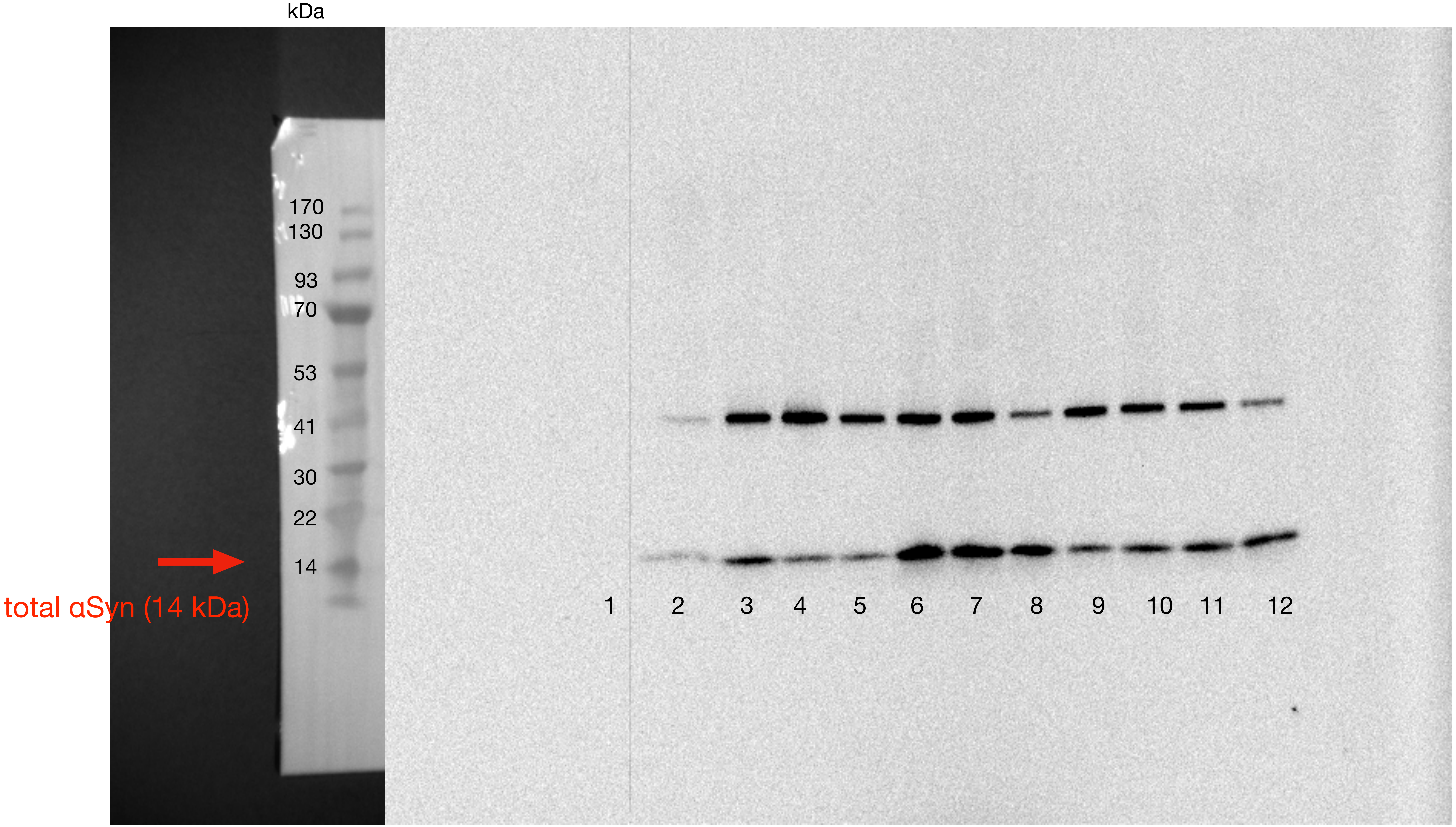

**1**	Marker
**2**	PBS+aSyn 1
**3**	PBS+aSyn 2
**4**	PBS+aSyn 3
**5**	PBS+aSyn 4
**6**	Vac+aSyn 1
**7**	Vac+aSyn 2
**8**	Vac+aSyn 3
**9**	Vac+aSyn 4
**10**	Vac+aSyn 5
**11**	Vac+aSyn 6
**12**	Vac+aSyn 7

Figure 5E: β-actin

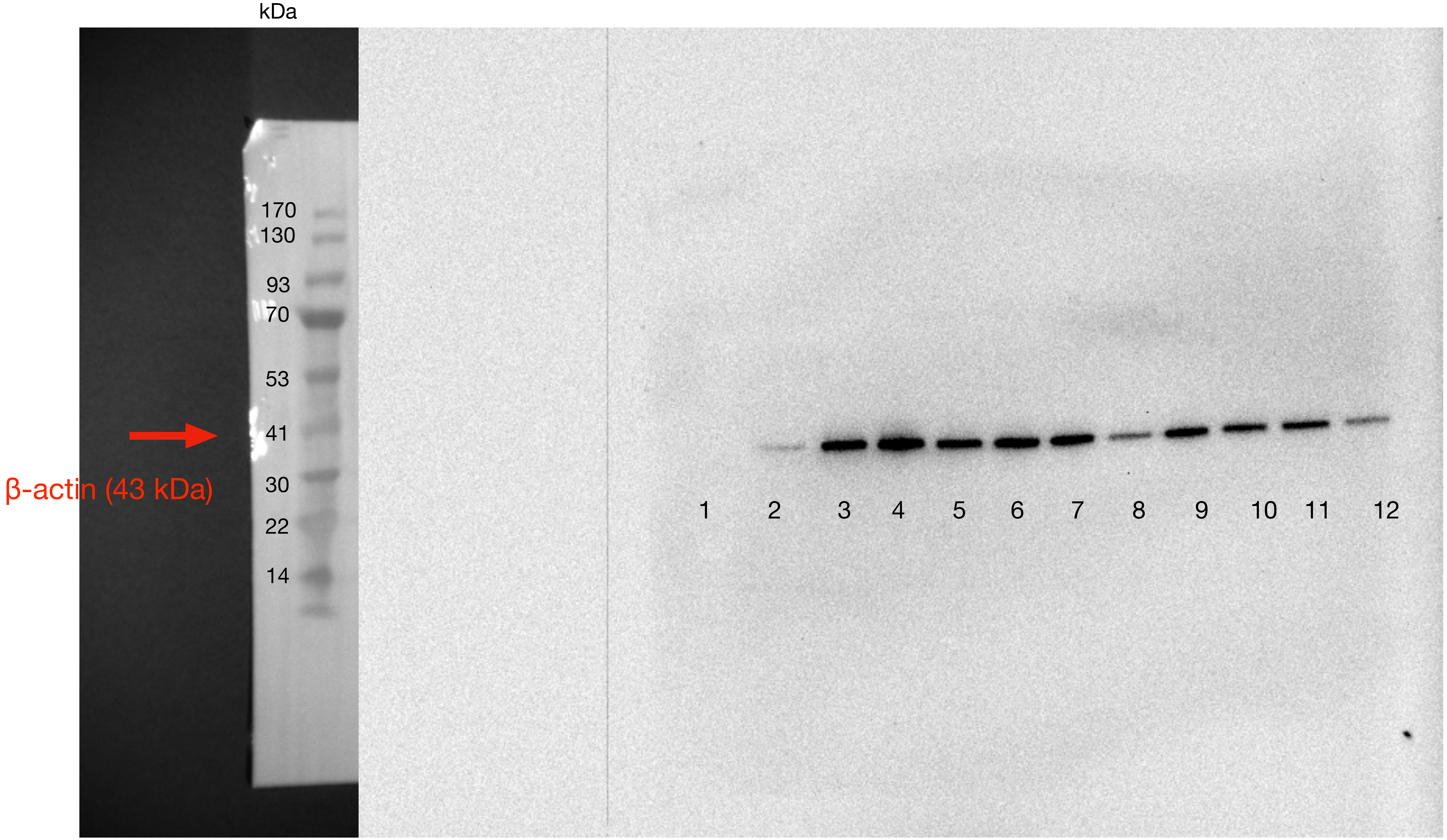

**1**	Marker
**2**	PBS+aSyn 1
**3**	PBS+aSyn 2
**4**	PBS+aSyn 3
**5**	PBS+aSyn 4
**6**	Vac+aSyn 1
**7**	Vac+aSyn 2
**8**	Vac+aSyn 3
**9**	Vac+aSyn 4
**10**	Vac+aSyn 5
**11**	Vac+aSyn 6
**12**	Vac+aSyn 7

Figure 5F: pS129-αSyn

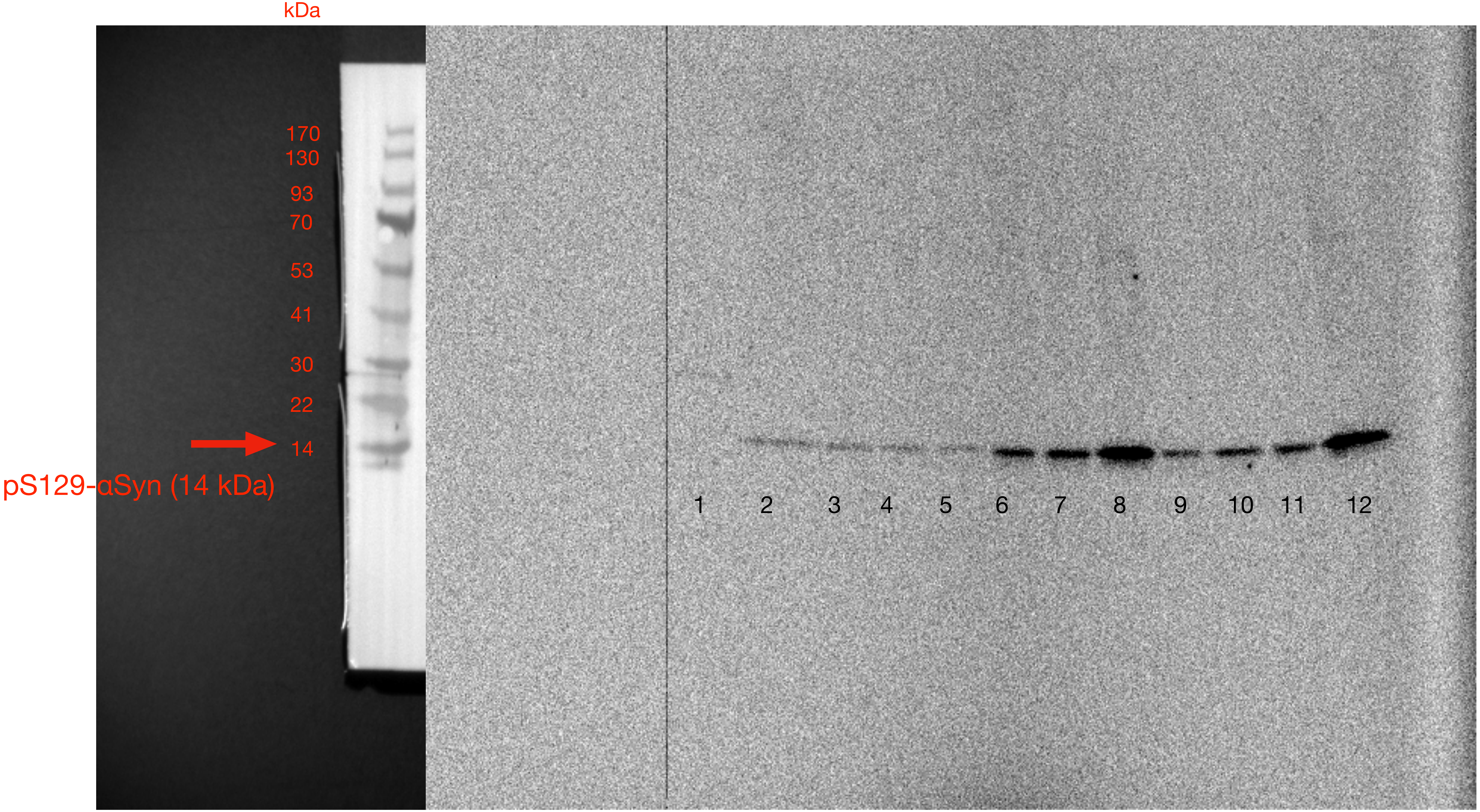

1	Marker
2	PBS+aSyn 1
3	PBS+aSyn 2
4	PBS+aSyn 3
5	PBS+aSyn 4
6	Vac+aSyn 1
7	Vac+aSyn 2
8	Vac+aSyn 3
9	Vac+aSyn 4
10	Vac+aSyn 5
11	Vac+aSyn 6
12	Vac+aSyn 7

Figure 5F: β-actin

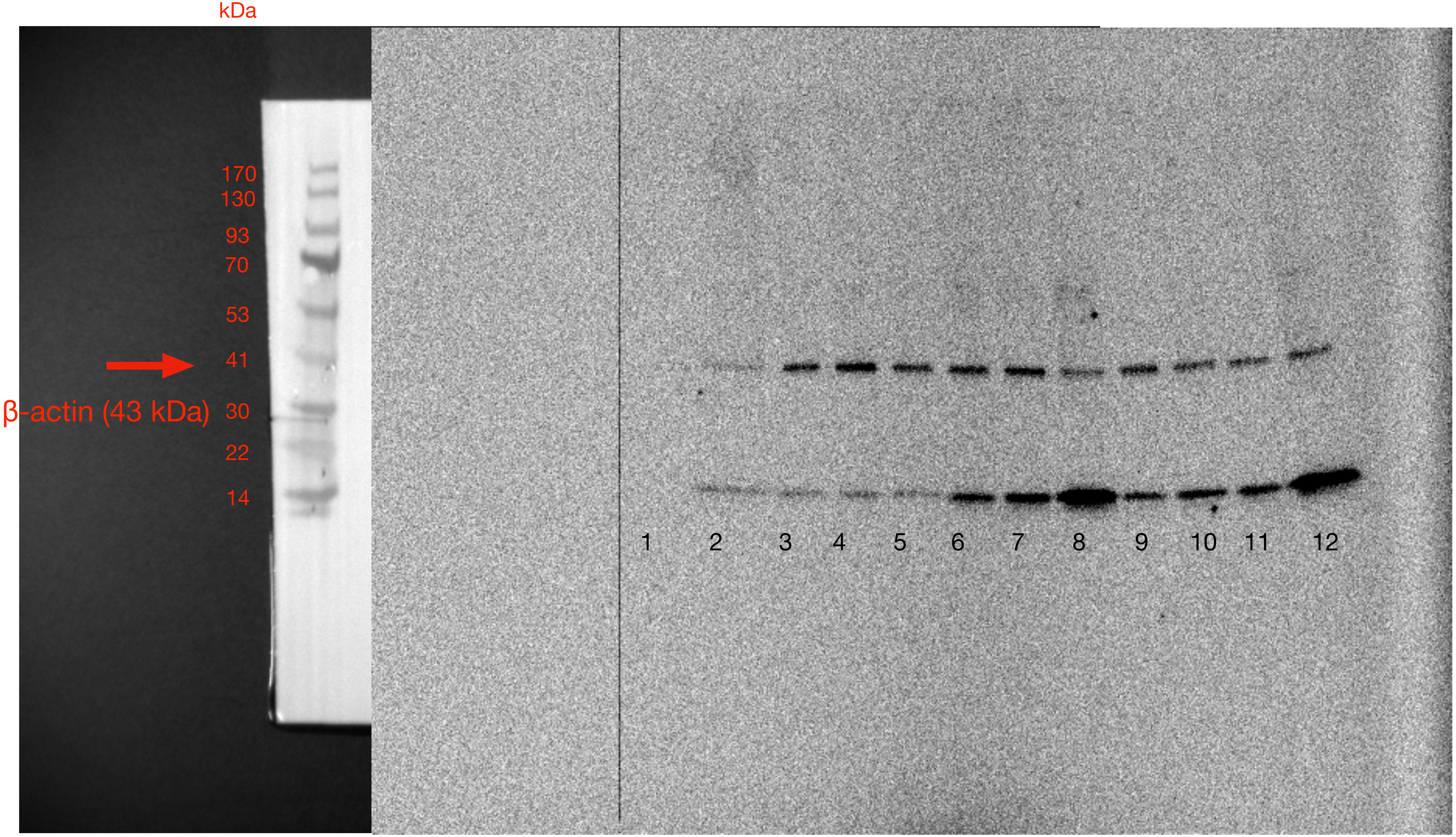

**1**	Marker
**2**	PBS+aSyn 1
**3**	PBS+aSyn 2
**4**	PBS+aSyn 3
**5**	PBS+aSyn 4
**6**	Vac+aSyn 1
**7**	Vac+aSyn 2
**8**	Vac+aSyn 3
**9**	Vac+aSyn 4
**10**	Vac+aSyn 5
**11**	Vac+aSyn 6
**12**	Vac+aSyn 7

Figure 6A: GFAP

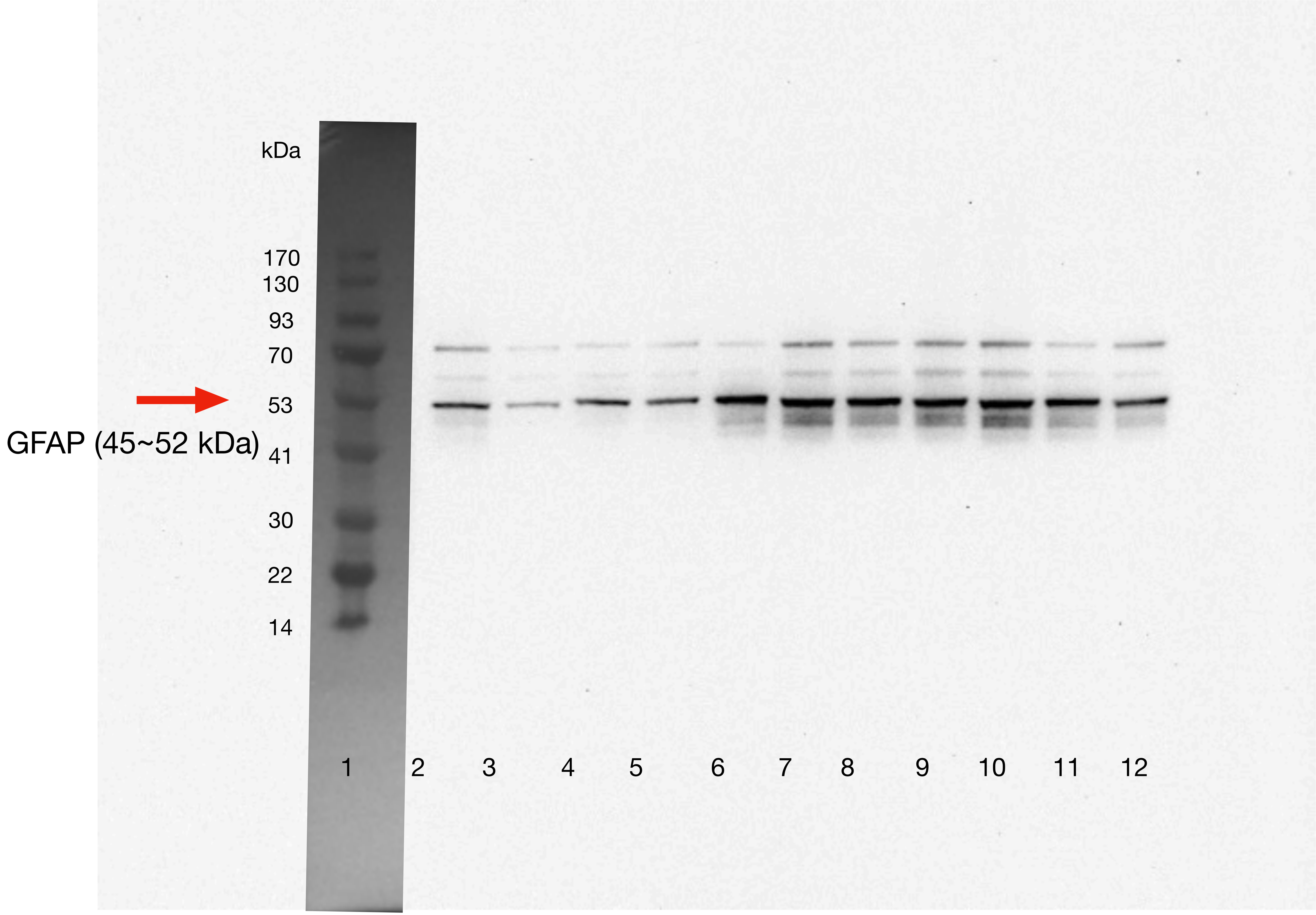

1	Marker
2	PBS+aSyn 1
3	PBS+aSyn 2
4	PBS+aSyn 3
5	PBS+aSyn 4
6	Vac+aSyn 1
7	Vac+aSyn 2
8	Vac+aSyn 3
9	Vac+aSyn 4
10	Vac+aSyn 5
11	Vac+aSyn 6
12	Vac+aSyn 7

Figure 6A: β-actin

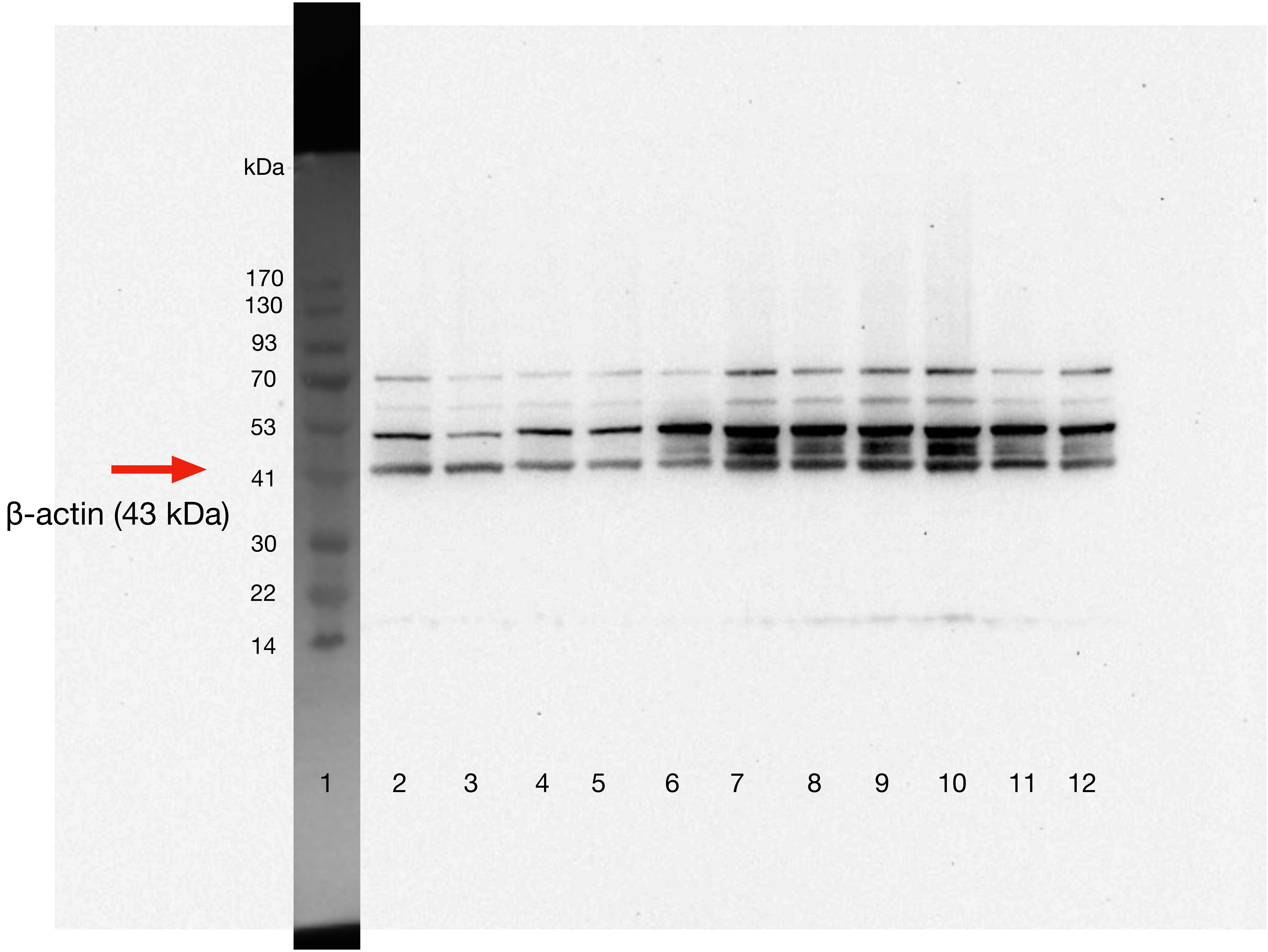

1	Marker
2	PBS+aSyn 1
3	PBS+aSyn 2
4	PBS+aSyn 3
5	PBS+aSyn 4
6	Vac+aSyn 1
7	Vac+aSyn 2
8	Vac+aSyn 3
9	Vac+aSyn 4
10	Vac+aSyn 5
11	Vac+aSyn 6
12	Vac+aSyn 7

Figure 6B: Iba1

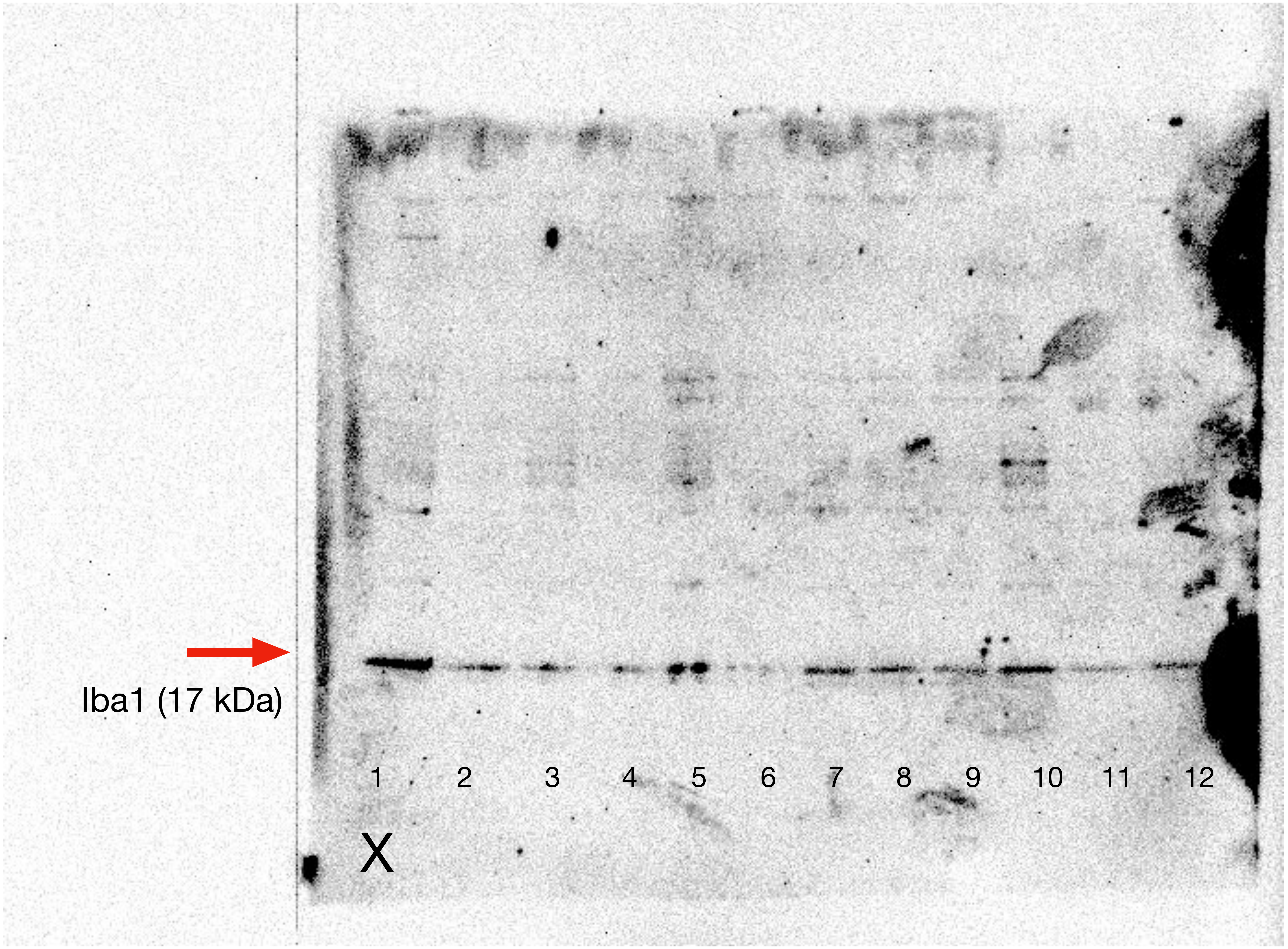

**1**	Iba1/b-actin positive control
**2**	PBS+aSyn 1
**3**	PBS+aSyn 2
**4**	PBS+aSyn 3
**5**	PBS+aSyn 4
**6**	Vac+aSyn 1
**7**	Vac+aSyn 2
**8**	Vac+aSyn 3
**9**	Vac+aSyn 4
**10**	Vac+aSyn 5
**11**	Vac+aSyn 6
**12**	Vac+aSyn 7

Figure 6B: β-actin

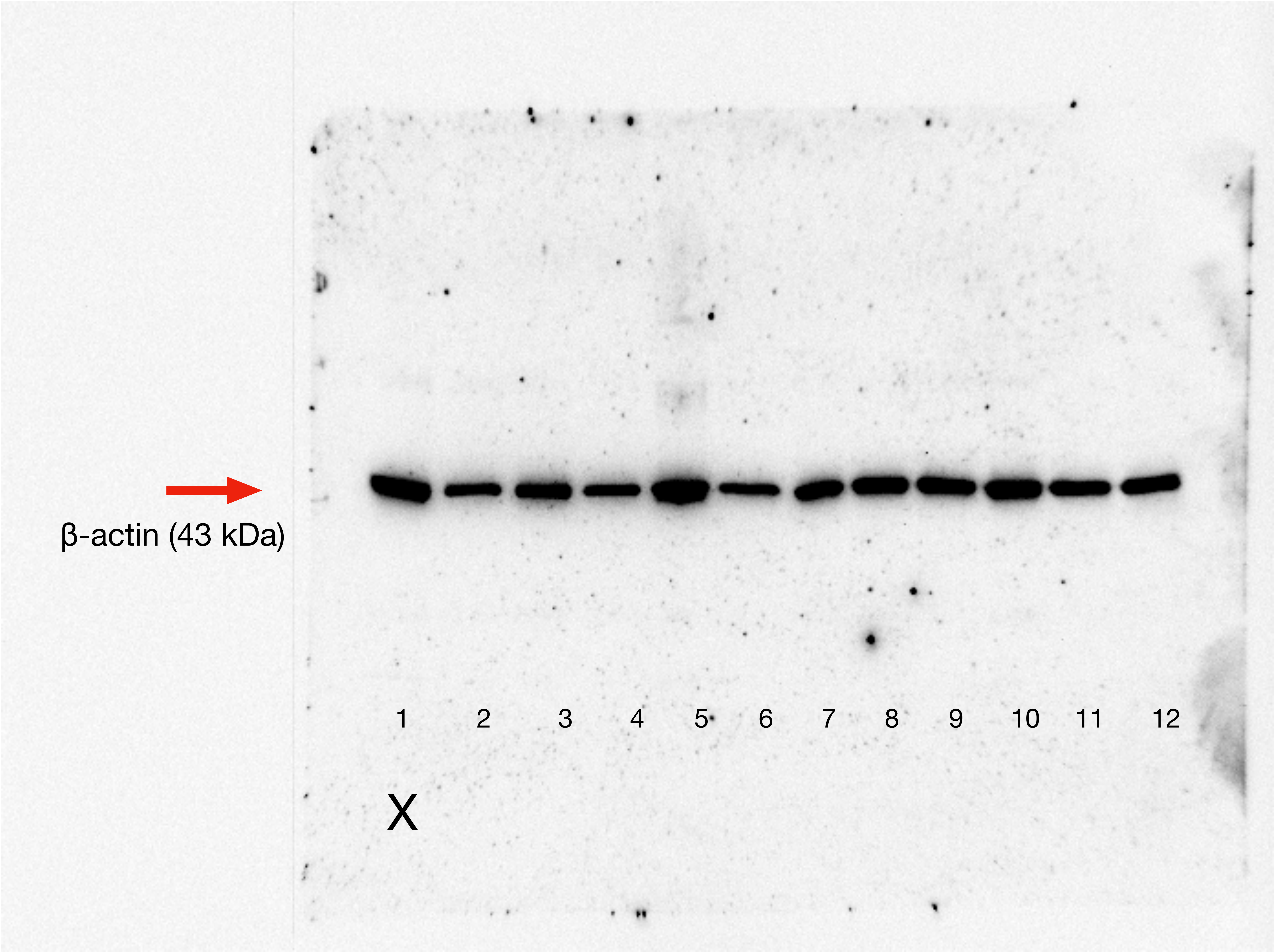

**1**	Iba1/b-actin positive control
**2**	PBS+aSyn 1
**3**	PBS+aSyn 2
**4**	PBS+aSyn 3
**5**	PBS+aSyn 4
**6**	Vac+aSyn 1
**7**	Vac+aSyn 2
**8**	Vac+aSyn 3
**9**	Vac+aSyn 4
**10**	Vac+aSyn 5
**11**	Vac+aSyn 6
**12**	Vac+aSyn 7

Figure 6C: TH

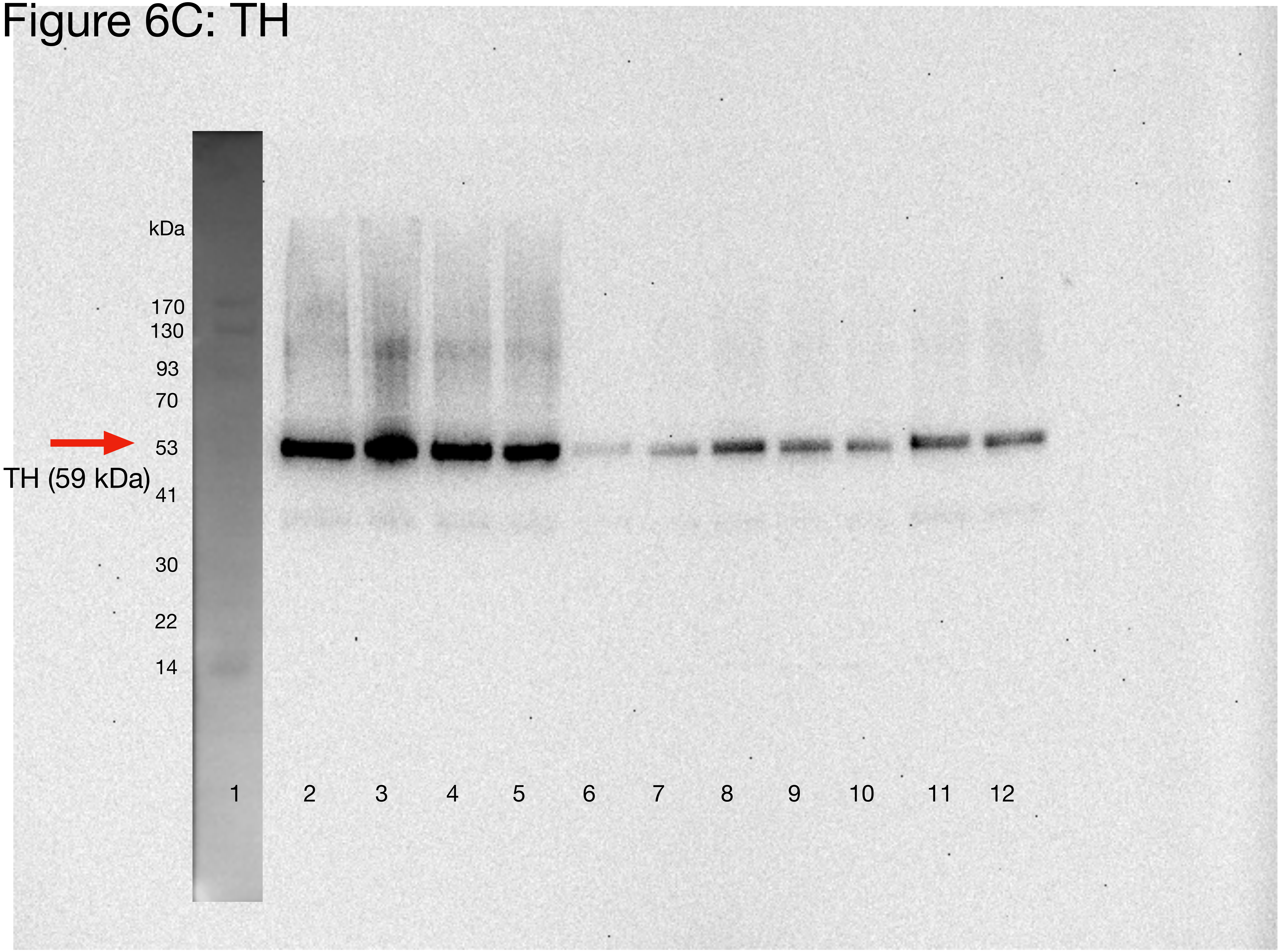

**1**	Marker
**2**	PBS+aSyn 1
**3**	PBS+aSyn 2
**4**	PBS+aSyn 3
**5**	PBS+aSyn 4
**6**	Vac+aSyn 1
**7**	Vac+aSyn 2
**8**	Vac+aSyn 3
**9**	Vac+aSyn 4
**10**	Vac+aSyn 5
**11**	Vac+aSyn 6
**12**	Vac+aSyn 7

Figure 6C: β-actin

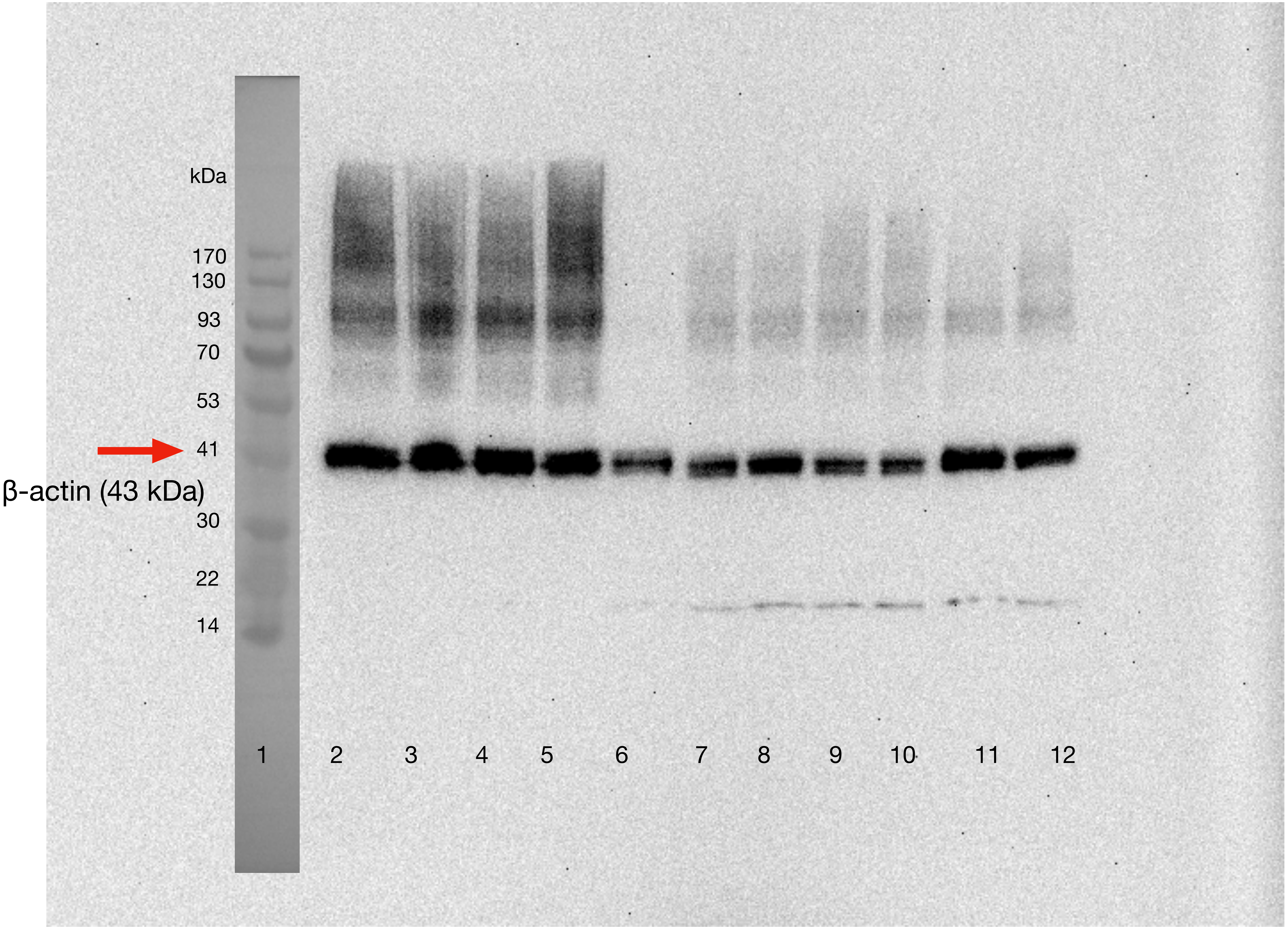

**1**	Marker
**2**	PBS+aSyn 1
**3**	PBS+aSyn 2
**4**	PBS+aSyn 3
**5**	PBS+aSyn 4
**6**	Vac+aSyn 1
**7**	Vac+aSyn 2
**8**	Vac+aSyn 3
**9**	Vac+aSyn 4
**10**	Vac+aSyn 5
**11**	Vac+aSyn 6
**12**	Vac+aSyn 7

Figure 6D: GFAP

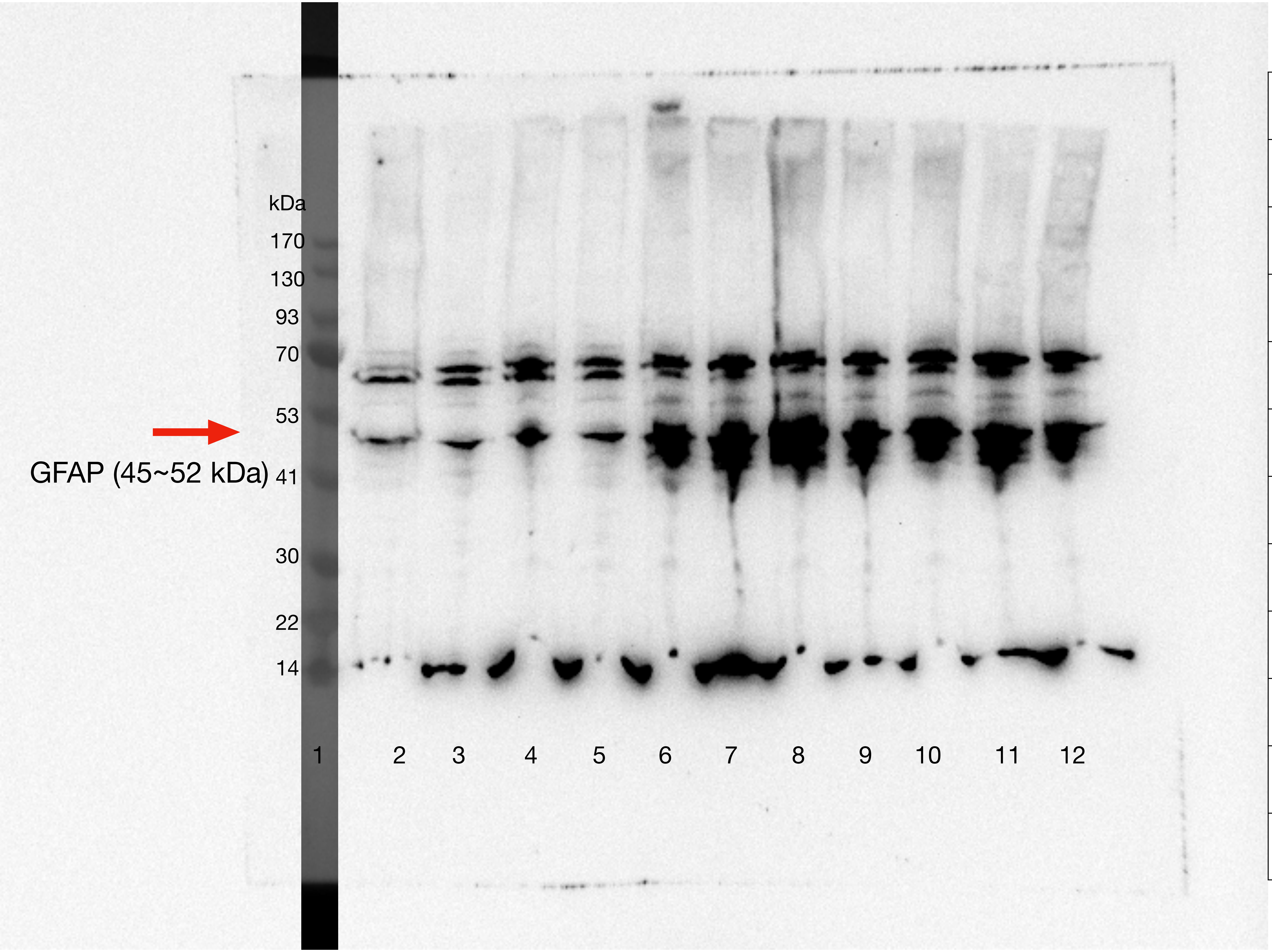

1	Marker
2	PBS+aSyn 1
3	PBS+aSyn 2
4	PBS+aSyn 3
5	PBS+aSyn 4
6	Vac+aSyn 1
7	Vac+aSyn 2
8	Vac+aSyn 3
9	Vac+aSyn 4
10	Vac+aSyn 5
11	Vac+aSyn 6
12	Vac+aSyn 7

Figure 6D: β-actin

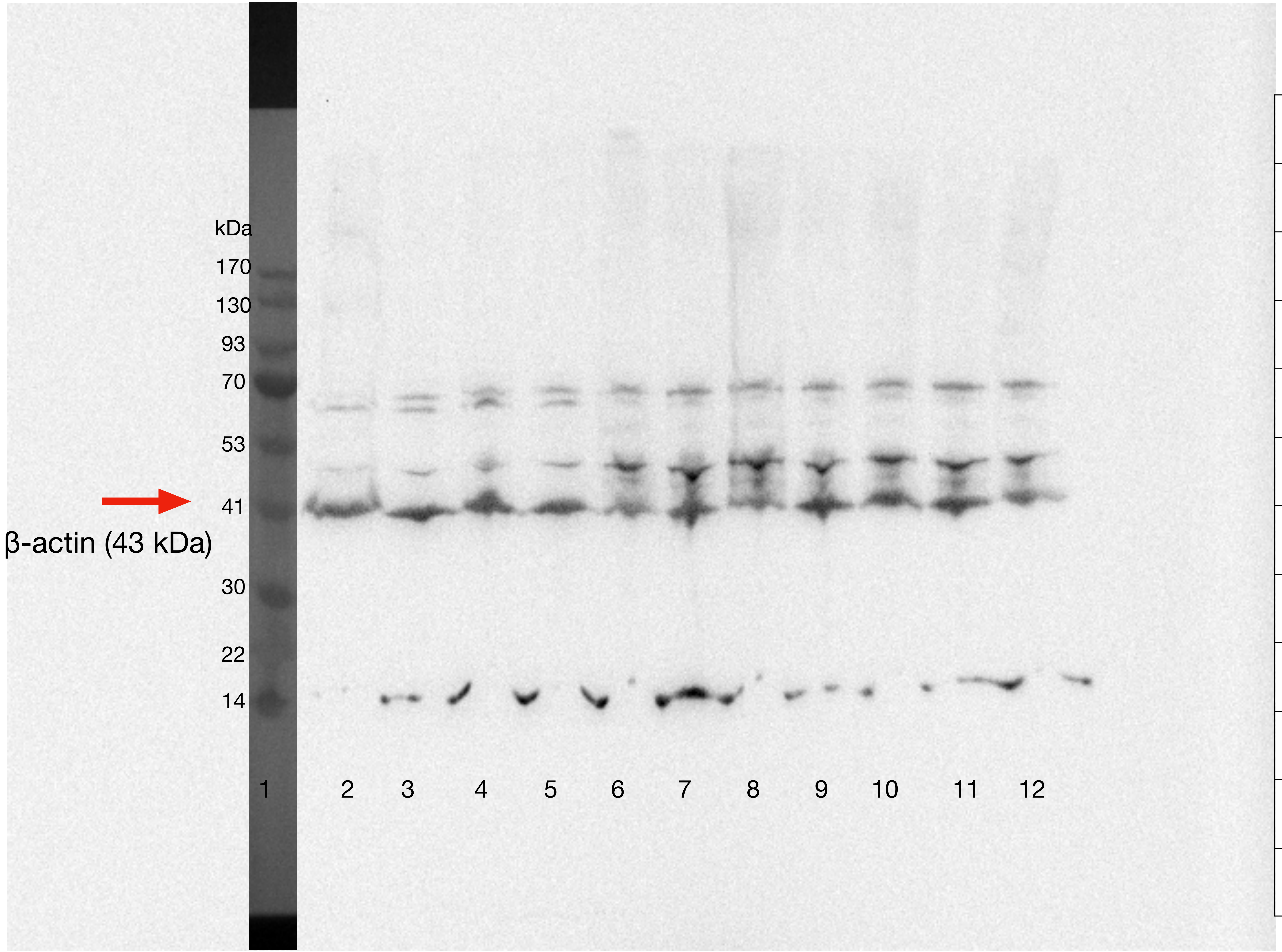

**1**	Marker
**2**	PBS+aSyn 1
**3**	PBS+aSyn 2
**4**	PBS+aSyn 3
**5**	PBS+aSyn 4
**6**	Vac+aSyn 1
**7**	Vac+aSyn 2
**8**	Vac+aSyn 3
**9**	Vac+aSyn 4
**10**	Vac+aSyn 5
**11**	Vac+aSyn 6
**12**	Vac+aSyn 7

Figure 6E: Iba1

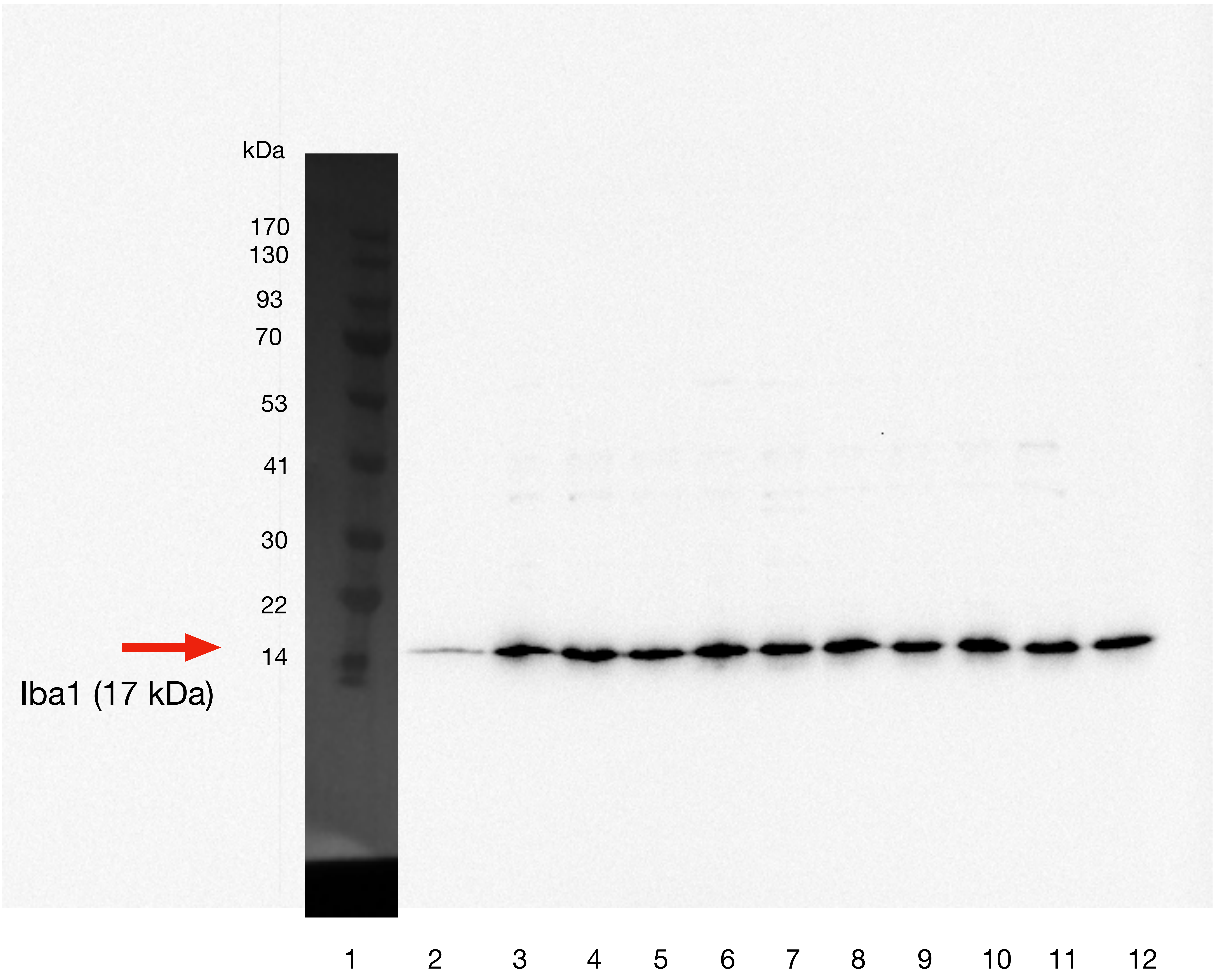

**1**	Marker
**2**	PBS+aSyn 1
**3**	PBS+aSyn 2
**4**	PBS+aSyn 3
**5**	PBS+aSyn 4
**6**	Vac+aSyn 1
**7**	Vac+aSyn 2
**8**	Vac+aSyn 3
**9**	Vac+aSyn 4
**10**	Vac+aSyn 5
**11**	Vac+aSyn 6
**12**	Vac+aSyn 7

Figure 6E: β-actin

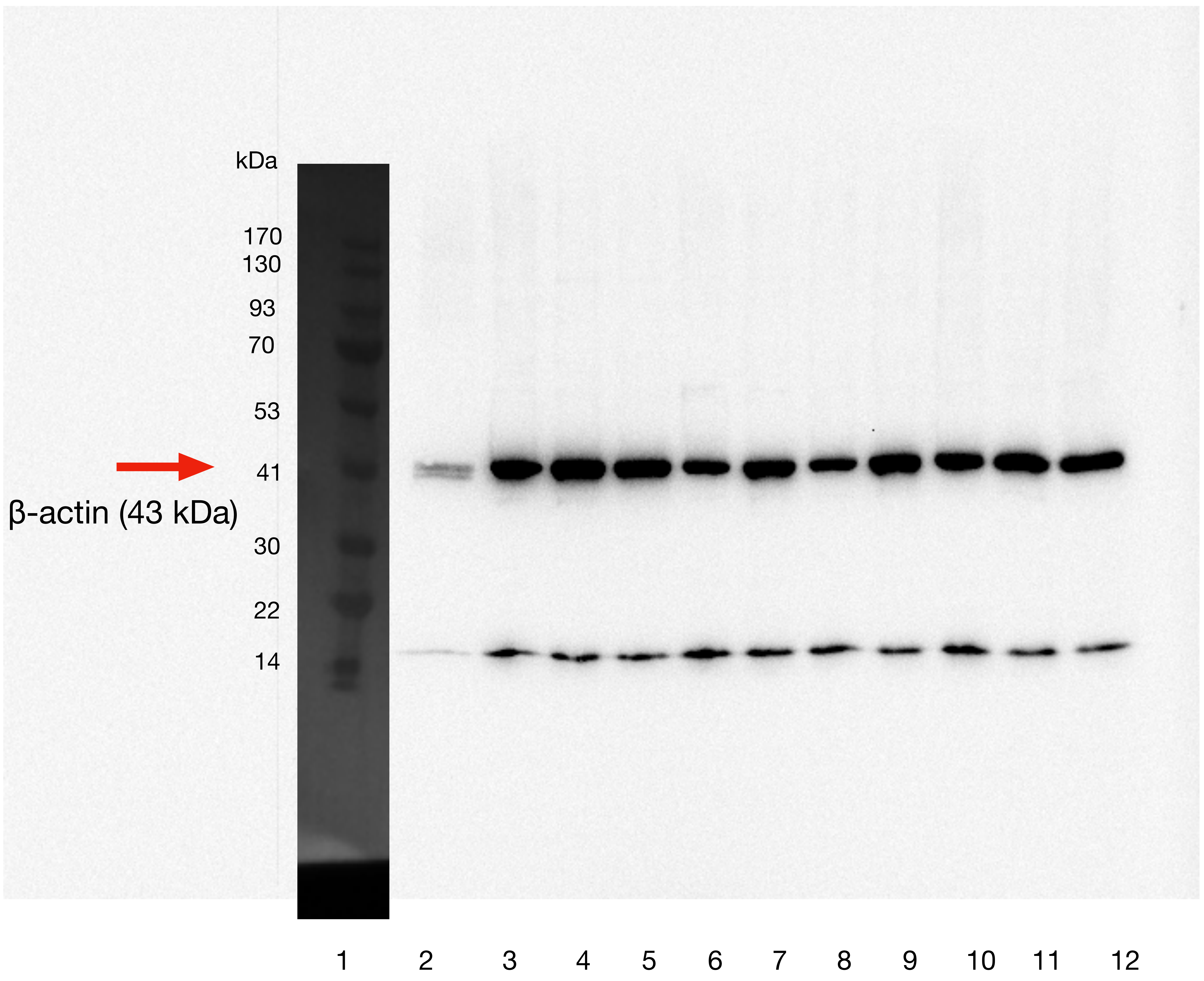

**1**	Marker
**2**	PBS+aSyn 1
**3**	PBS+aSyn 2
**4**	PBS+aSyn 3
**5**	PBS+aSyn 4
**6**	Vac+aSyn 1
**7**	Vac+aSyn 2
**8**	Vac+aSyn 3
**9**	Vac+aSyn 4
**10**	Vac+aSyn 5
**11**	Vac+aSyn 6
**12**	Vac+aSyn 7

Figure 6F: TH

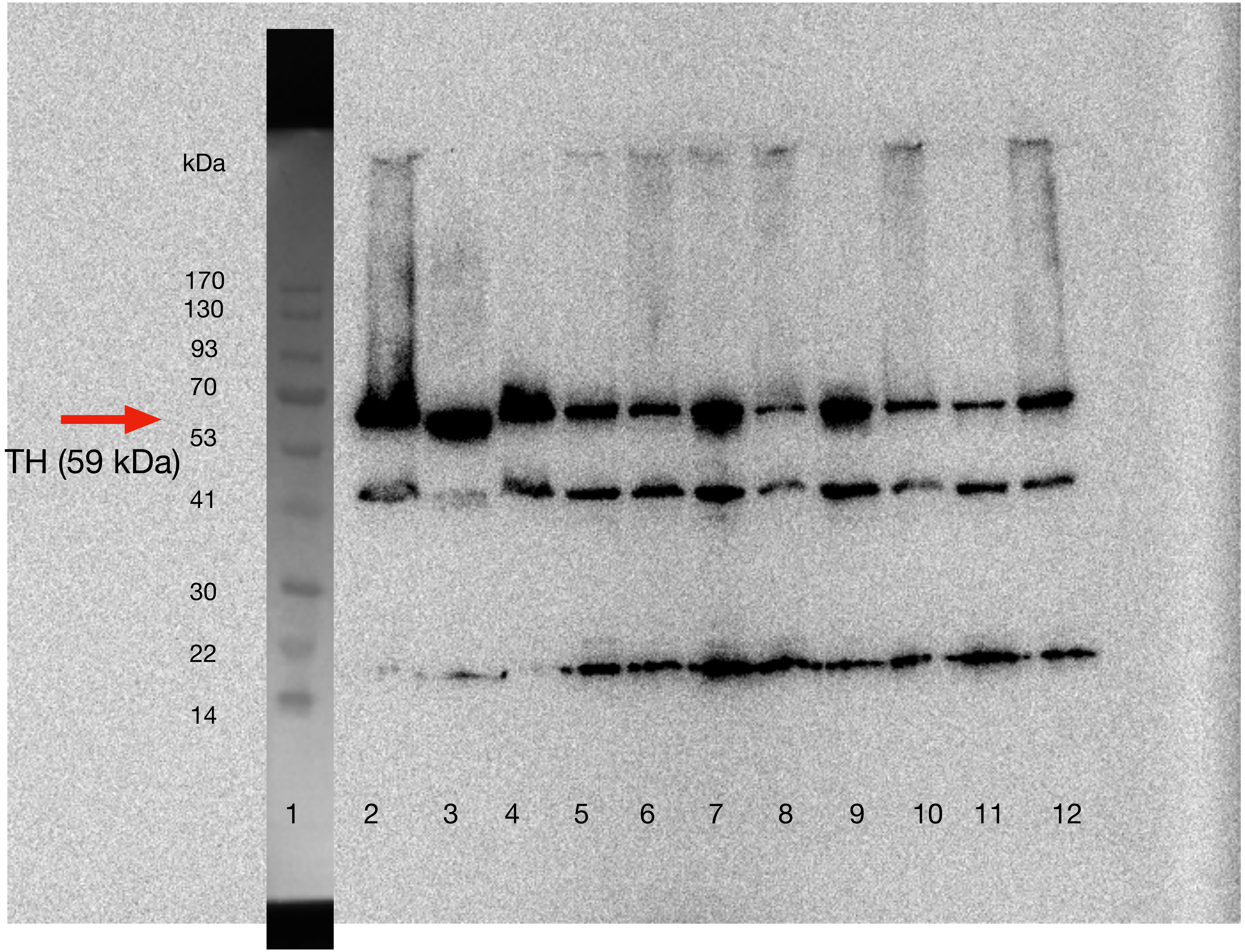

**1**	Marker
**2**	PBS+aSyn 1
**3**	PBS+aSyn 2
**4**	PBS+aSyn 3
**5**	PBS+aSyn 4
**6**	Vac+aSyn 1
**7**	Vac+aSyn 2
**8**	Vac+aSyn 3
**9**	Vac+aSyn 4
**10**	Vac+aSyn 5
**11**	Vac+aSyn 6
**12**	Vac+aSyn 7

Figure 6F: β-actin

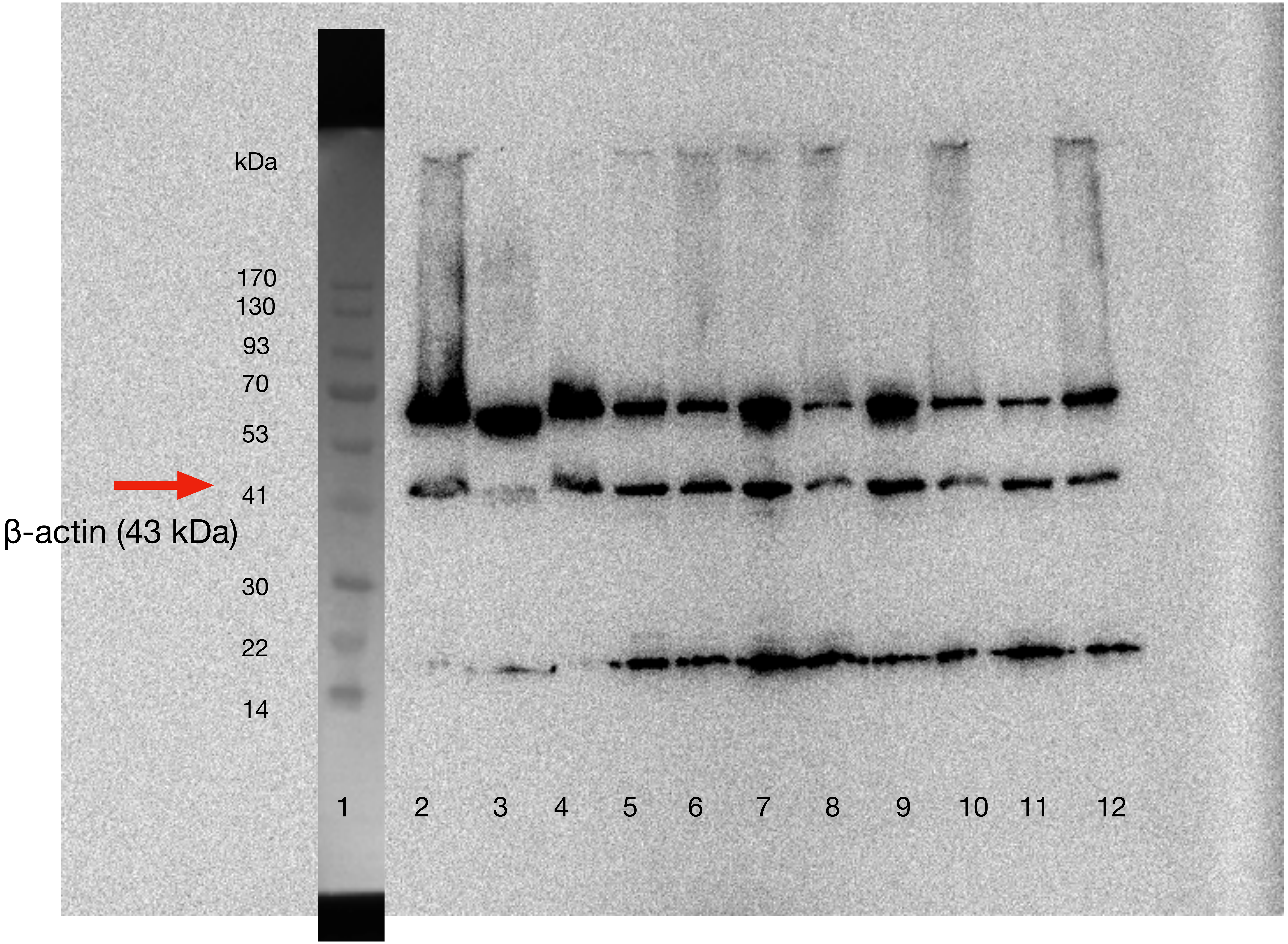

**1**	Marker
**2**	PBS+aSyn 1
**3**	PBS+aSyn 2
**4**	PBS+aSyn 3
**5**	PBS+aSyn 4
**6**	Vac+aSyn 1
**7**	Vac+aSyn 2
**8**	Vac+aSyn 3
**9**	Vac+aSyn 4
**10**	Vac+aSyn 5
**11**	Vac+aSyn 6
**12**	Vac+aSyn 7

Figure 7A: Synaptophysin

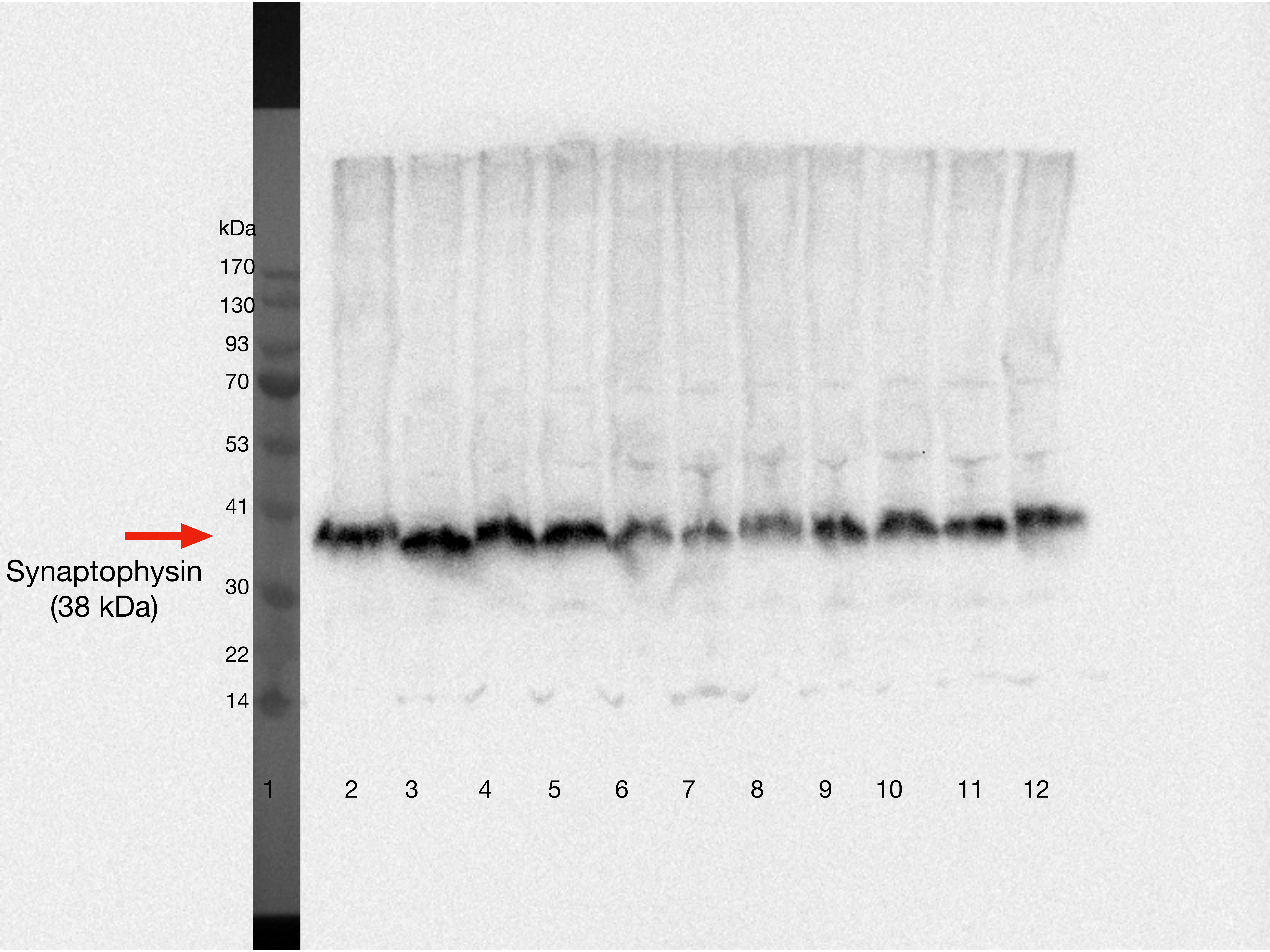

**1**	Marker
**2**	PBS+aSyn 1
**3**	PBS+aSyn 2
**4**	PBS+aSyn 3
**5**	PBS+aSyn 4
**6**	Vac+aSyn 1
**7**	Vac+aSyn 2
**8**	Vac+aSyn 3
**9**	Vac+aSyn 4
**10**	Vac+aSyn 5
**11**	Vac+aSyn 6
**12**	Vac+aSyn 7

Figure 7A: β-actin

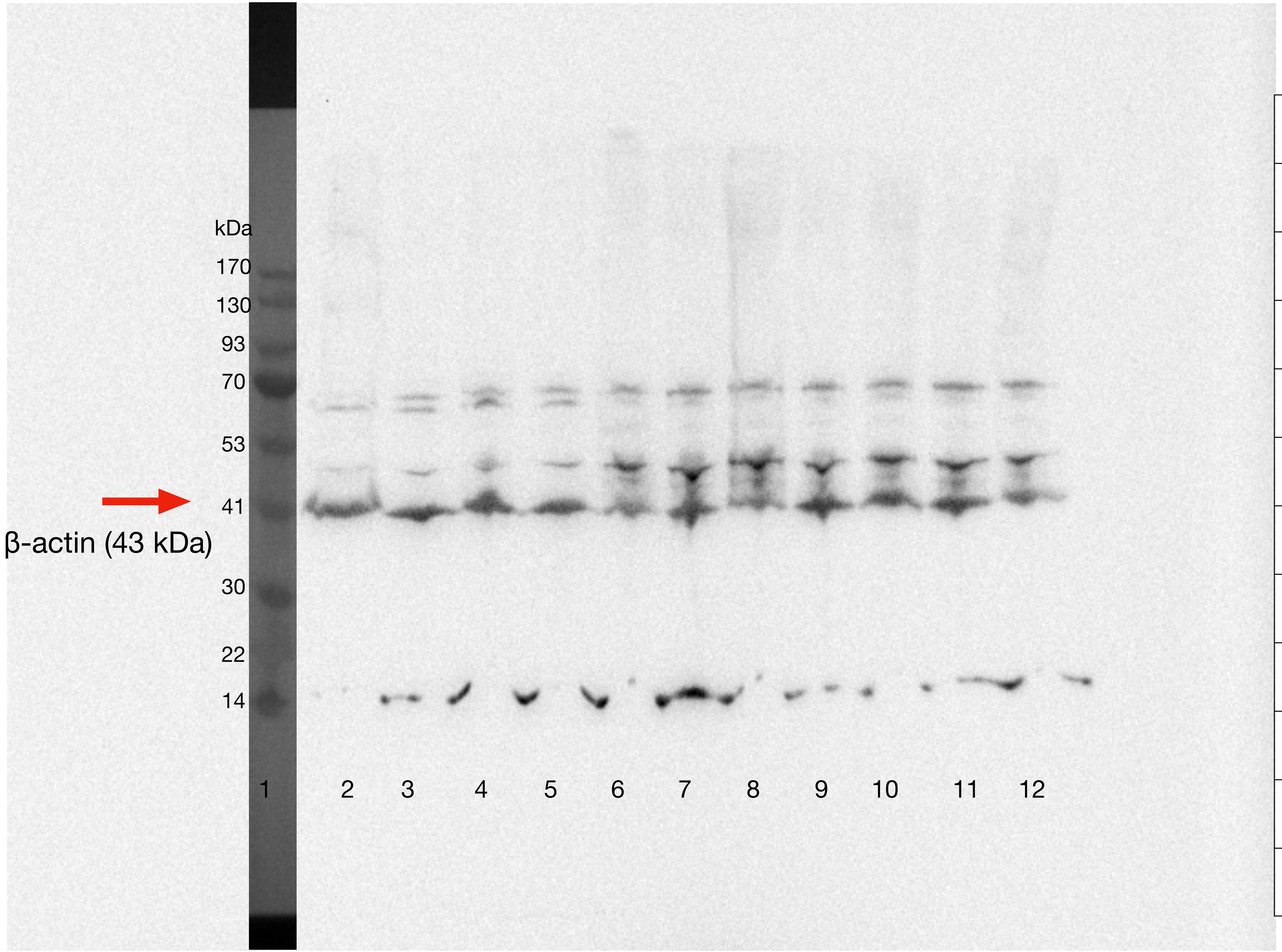

**1**	Marker
**2**	PBS+aSyn 1
**3**	PBS+aSyn 2
**4**	PBS+aSyn 3
**5**	PBS+aSyn 4
**6**	Vac+aSyn 1
**7**	Vac+aSyn 2
**8**	Vac+aSyn 3
**9**	Vac+aSyn 4
**10**	Vac+aSyn 5
**11**	Vac+aSyn 6
**12**	Vac+aSyn 7

Figure 7B: PSD95

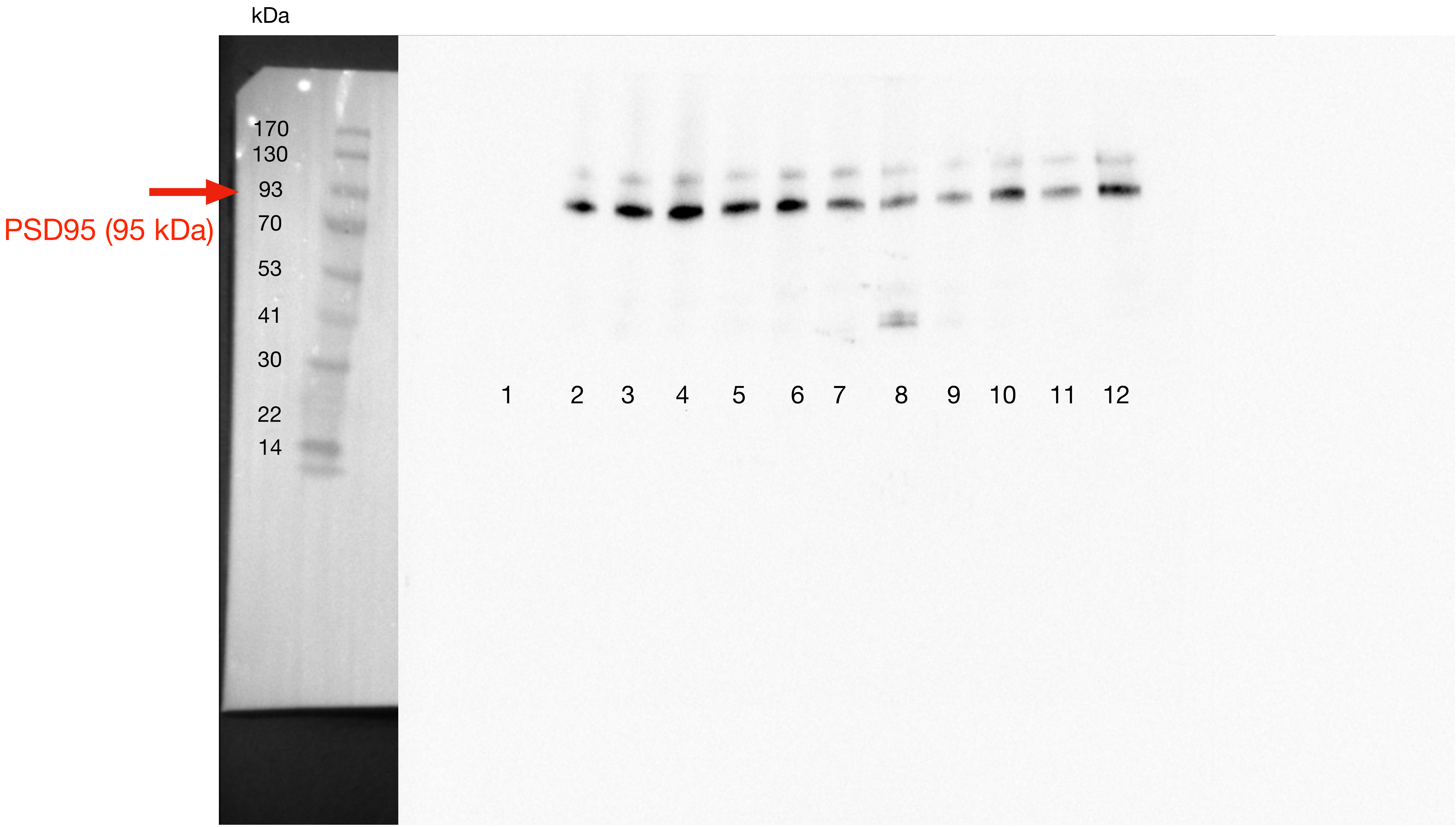

**1**	Marker
**2**	PBS+aSyn 1
**3**	PBS+aSyn 2
**4**	PBS+aSyn 3
**5**	PBS+aSyn 4
**6**	Vac+aSyn 1
**7**	Vac+aSyn 2
**8**	Vac+aSyn 3
**9**	Vac+aSyn 4
**10**	Vac+aSyn 5
**11**	Vac+aSyn 6
**12**	Vac+aSyn 7

Figure 7B: β-actin

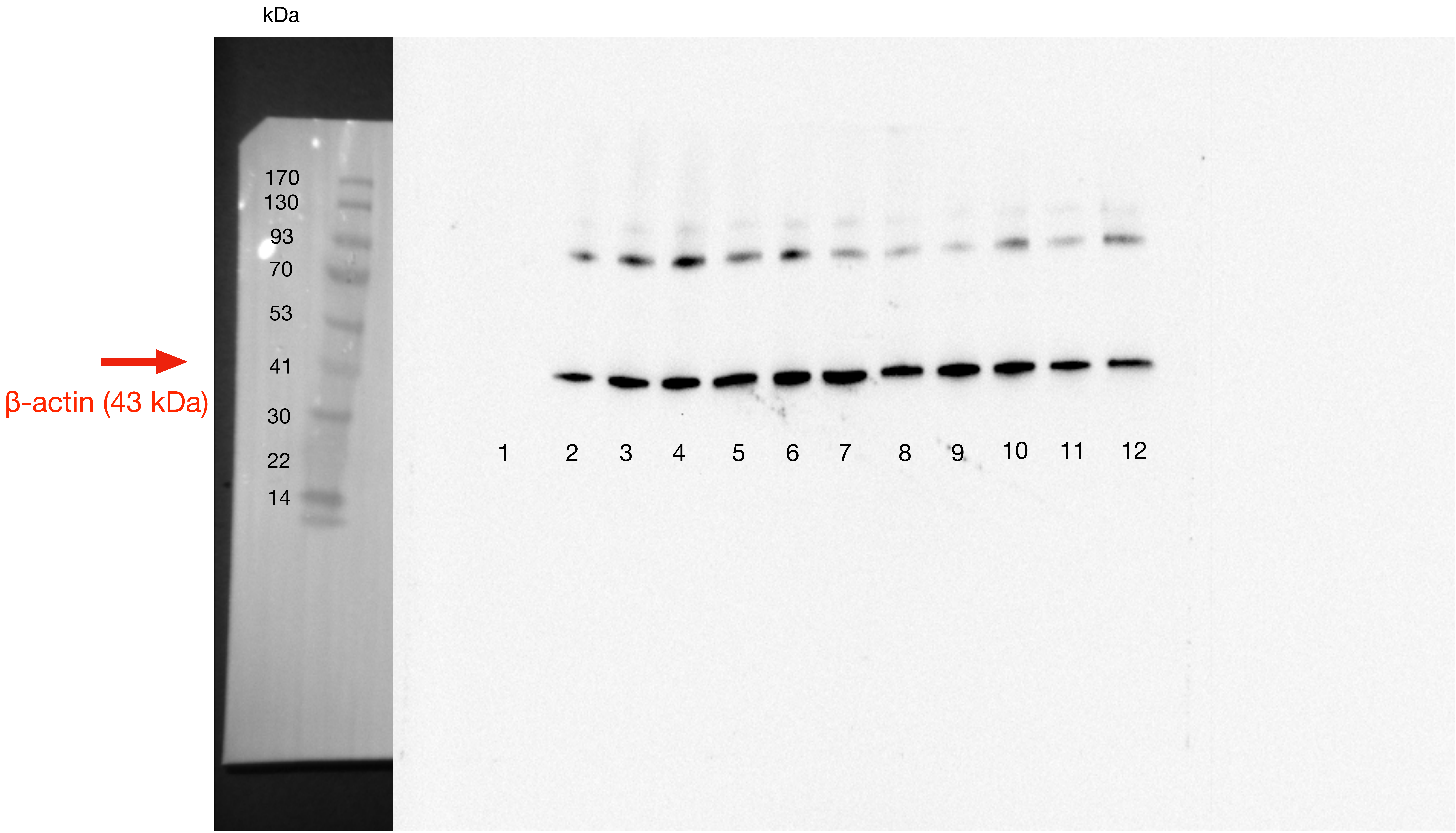

**1**	Marker
**2**	PBS+aSyn 1
**3**	PBS+aSyn 2
**4**	PBS+aSyn 3
**5**	PBS+aSyn 4
**6**	Vac+aSyn 1
**7**	Vac+aSyn 2
**8**	Vac+aSyn 3
**9**	Vac+aSyn 4
**10**	Vac+aSyn 5
**11**	Vac+aSyn 6
**12**	Vac+aSyn 7
